# Vasculogenic mimicry in small cell lung cancer

**DOI:** 10.1038/ncomms13322

**Published:** 2016-11-09

**Authors:** Stuart C. Williamson, Robert L. Metcalf, Francesca Trapani, Sumitra Mohan, Jenny Antonello, Benjamin Abbott, Hui Sun Leong, Christopher P. E. Chester, Nicole Simms, Radoslaw Polanski, Daisuke Nonaka, Lynsey Priest, Alberto Fusi, Fredrika Carlsson, Anders Carlsson, Mary J. C. Hendrix, Richard E. B. Seftor, Elisabeth A. Seftor, Dominic G. Rothwell, Andrew Hughes, James Hicks, Crispin Miller, Peter Kuhn, Ged Brady, Kathryn L. Simpson, Fiona H. Blackhall, Caroline Dive

**Affiliations:** 1 Clinical and Experimental Pharmacology Group, Cancer Research UK Manchester Institute, Manchester M20 4BX, UK; 2 Computational Biology Support Team, Cancer Research UK Manchester Institute, Manchester M20 4BX, UK; 3 The Christie NHS Foundation Trust, Manchester M20 4BX, UK; 4 University of Southern California Dornsife, Los Angeles, California 90089-3301, USA; 5 Stanley Manne Children's Research Institute, Robert H. Lurie Comprehensive Cancer Center, Northwestern University Feinberg School of Medicine, Chicago, Illinois 60611, USA; 6 The Institute of Cancer Sciences, University of Manchester, Manchester M20 4BX, UK; 7 RNA Biology Group, Cancer Research UK Manchester Institute, Manchester M20 4BX, UK; 8 Cancer Research UK, Lung Cancer Centre of Excellence, Manchester M20 4BX, UK

## Abstract

Small cell lung cancer (SCLC) is characterized by prevalent circulating tumour cells (CTCs), early metastasis and poor prognosis. We show that SCLC patients (37/38) have rare CTC subpopulations co-expressing vascular endothelial-cadherin (VE-cadherin) and cytokeratins consistent with vasculogenic mimicry (VM), a process whereby tumour cells form ‘endothelial-like' vessels. Single-cell genomic analysis reveals characteristic SCLC genomic changes in both VE-cadherin-positive and -negative CTCs. Higher levels of VM are associated with worse overall survival in 41 limited-stage patients' biopsies (*P*<0.025). VM vessels are also observed in 9/10 CTC patient-derived explants (CDX), where molecular analysis of fractionated VE-cadherin-positive cells uncovered copy-number alterations and mutated *TP53,* confirming human tumour origin. VE-cadherin is required for VM in NCI-H446 SCLC xenografts, where VM decreases tumour latency and, despite increased cisplatin intra-tumour delivery, decreases cisplatin efficacy. The functional significance of VM in SCLC suggests VM regulation may provide new targets for therapeutic intervention.

## Abstract (web summary)

Small cell lung cancer (SCLC) is characterised by prevalent circulating tumour cells (CTCs), early metastasis and poor prognosis. The authors show that SCLC patients have a rare CTC subset with a vasculogenic mimicry (VM) phenotype, and that VM is associated with worse overall survival, and alters tumour growth, chemotherapy delivery and efficacy.

## Graphical abstract

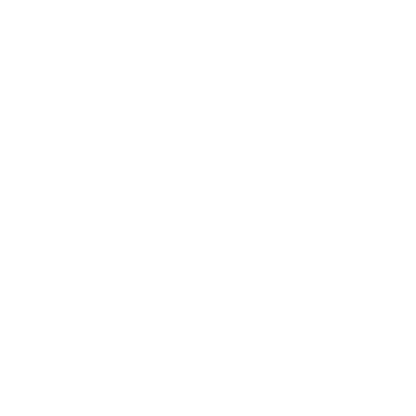

Small cell lung cancer (SCLC) accounts for ∼15% of lung cancer cases, ∼200,000 deaths worldwide per annum and is characterized by early dissemination^1^. Approximately 33% of SCLC patients present with cancer confined to one hemithorax, referred to as limited-stage (LS) disease. However, the majority of patients present with Extensive-Stage (ES) disease with involvement of lymph nodes and with distant metastases^2^. Most patients initially respond to platinum-based chemotherapy, but usually relapse and acquire chemo-resistant disease^3^ such that the median overall survival (OS) is 9–12 months and 5-year survival is <5% (ref. 4). Advances with targeted therapies are yet to materialise and chemotherapy regimens have remained unchanged for almost three decades^5^. Improved understanding of SCLC biology is essential to instruct development of improved treatments, for example, by targeting SCLC stem cells^6^ or overcoming mechanisms of chemotherapy resistance.

First reported in uveal melanoma^7^, vasculogenic mimicry (VM) describes the ability of aggressive tumour cells to trans-differentiate, acquiring endothelial cell behaviour that enables *de novo* generation of tumour-derived vascular networks and a micro-circulation that is independent of non-cancer host cells^8^. VM is evaluated in clinical specimens by immunohistochemical (IHC) analysis of Periodic Acid Schiff (PAS) positive, CD31 negative vessels^9^. PAS stains basement membranes including laminin, collagen and glycogen^10^ and CD31 is an endothelial cell marker. A systematic review with meta-analysis of VM, including 3,062 patients with multiple cancer types, showed that VM was positively correlated with worse 5-year OS^11^ and VM is linked to promotion of aggressive disease and metastatic spread^12^. The transcriptional signature of VM^13^,^14^,^15^ shares components with that of ‘stemness' and Epithelial-to-Mesenchymal Transition (EMT), key attributes involving tumour plasticity during metastasis and resistance to chemotherapy^16^. Furthermore, expression of ‘stem' cell markers was associated with VM in Ewing's sarcoma^17^ and in a subpopulation of breast cancer cells^18^. Key molecular regulators of VM identified in other cancer types, include a VM-related anti-coagulant role for Serpine2 and Slpi in a preclinical breast cancer model^12^. Transmembrane proteins involved in adhesion and cellular polarity are upregulated in aggressive VM-forming cells, including Claudin-4, required for formation and maintenance of endothelial and epithelial tight junctions in breast cancer cells^19^ and Vascular Endothelial (VE)-Cadherin, a transmembrane cell adhesion molecule identified as a key regulator of VM in aggressive melanoma^20^. There are no previous reports of VM in SCLC, although it was described in Merkel cell carcinoma, which, like SCLC, is of neuroendocrine origin^21^.

We argue that if VM contributes to tumour cell dissemination in patients, then the VM phenotype is likely to be represented within circulating tumour cells (CTCs), as tumour cells forming vessel walls have easy access to the bloodstream. We previously reported the high prevalence and prognostic significance of CTCs in SCLC^22^ and for the first time we report the expression of VM-associated VE-cadherin in a rare subset of SCLC CTCs. We also demonstrate the presence of VM in SCLC patient tissues, CTC patient-derived explant (CDX) models^23^ and a VE-cadherin expressing SCLC xenograft. Given previous controversy surrounding the tumour origin of VM cells^24^,^25^,^26^, we sought genomic verification that SCLC cells, rather than endothelial cells, were responsible for observed VM phenotypes. Finally, we asked whether VE-cadherin has functional significance in SCLC with respect to tumour growth kinetics, the intra-tumoural delivery and efficacy of cisplatin (used together with etoposide as SCLC standard of care). We hypothesize that *in vivo*, VM may promote opposing impacts on tumours; on the one hand VM may aid drug and nutrient delivery, on the other, it may endow an aggressive, drug resistant, ‘stem-like' phenotype that could contribute to dissemination, metastasis, disease relapse and shortened patient survival in this dismal disease^22^.

## Results

### A rare subset of SCLC CTCs exhibit a VM phenotype

**Figure 1:**
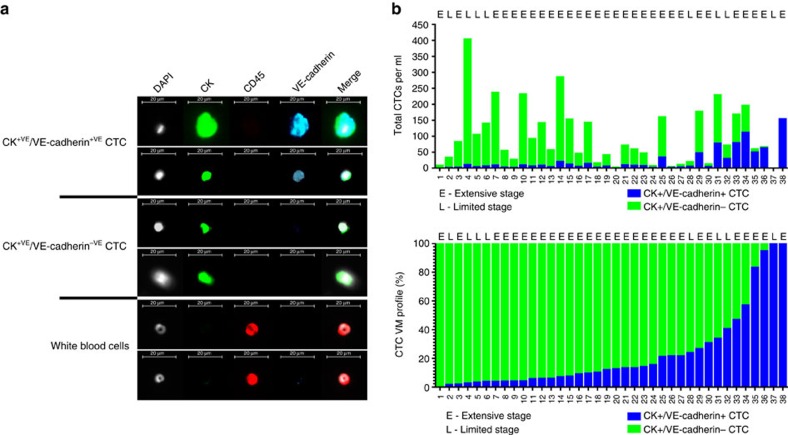
ISET-filtered CTCs from patients with either LS or ES SCLC co-express VE-cadherin and CKs. (**a**) Pseudo-coloured ISET filters stained with DAPI (white) and antibodies to pan-CK (green), CD45 (red) and VE-cadherin (blue). CTCs are classified as DAPI^+VE^/CD45^−VE^/CK^+VE^. Scale bar, 20 μm. (**b**) Top panel, total CTC counts per ml for CK^+VE^/VE-cadherin^+VE^ CTCs (blue bars) versus CK^+VE^/VE-cadherin^−VE^ CTCs (green bars) are shown for patients 1 to 38. (**b**) Bottom panel, the percentage of CK^+VE^/VE-cadherin^+VE^ CTCs (blue bars) versus CK^+VE^/VE-cadherin^−VE^ CTCs (green bars) is shown for patients 1–38.

We hypothesized that if SCLC cells line VM vessels, then these cells would have ready access into the circulation. Using VE-cadherin as a VM biomarker^20^, we assessed its expression in SCLC cytokeratin (CK)-positive CTCs using ISET (isolation by size of epithelial tumour) microfiltration of patient blood samples that captures cells larger than 8 μm (median SCLC cell size 12.5–14.1 μm (ref. 27); Fig. 1). Thirty eight chemo-naïve SCLC patients were consented to a prospective biomarker programme for the investigation of blood-borne biomarkers in lung cancer, conducted at the Christie NHS Foundation Trust, Manchester, United Kingdom. Each patient had DAPI^+VE^/pan-CK, CK^+VE^/CD45^−VE^ CTCs (Fig. 1a and Supplementary Fig. 1a) that ranged from 0.25–403, (median 68, mean 100) CTCs per ml. All but 1 of these patients (patient 1) exhibited a rare subpopulation of DAPI^+VE^/CK^+VE^/VE-cadherin^+VE^ CTCs (Fig. 1a and Supplementary Fig. 1). DAPI^+VE^/CK^+VE^/VE-cadherin^+VE^ CTC number ranged from 0–154 (median 6, mean 21) CTCs per ml (Fig. 1b, upper panel) highlighting the challenge of identifying this CTC subpopulation. The proportion of VE-cadherin expressing CTCs ranged from 0–100% (median 12%, mean 23%) of all DAPI^+VE^/CK^+VE^ CTCs (Fig. 1b lower panel).

For 24 of the ES SCLC patients' blood samples assessed by ISET, we were able to access a matched needle-core biopsy in which to evaluate VM using PAS and anti-human anti-CD31 IHC. However, the poor quality of these biopsies (especially crush artefact^28^), combined with the low percentage of tumour content, made it impossible to assess VM robustly in this type of sample and an alternative source of SCLC tumour samples was sought to assess VM.

### VM is present and prognostic in LS SCLC

**Figure 2:**
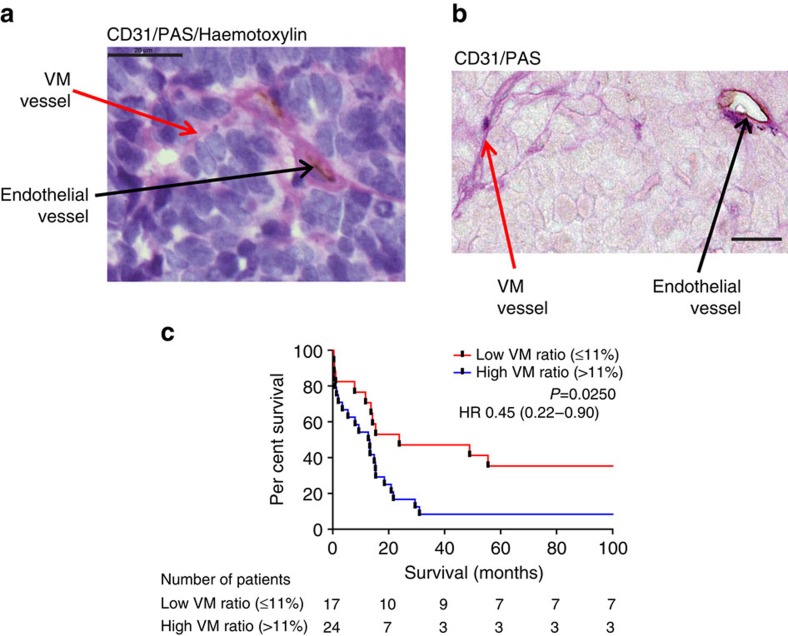
Vasculogenic Mimicry in metastatic lymph nodes from LS SCLC patients and associated patient overall survival. One-milimetre cores taken during lymph node resection were stained with anti-human anti-CD31 (brown), periodic acid schiff (PAS; pink) with or without haematoxylin (purple). (**a**) Region showing typical malignant morphology of the SCLC; PAS^+VE^/CD31^+VE^ blood vessels (black arrow) and PAS^+VE^/CD31^−VE^ VM vessels (red arrow; × 100 magnification). (**b**) Loops and whorls characteristic of VM via light microscopy ( × 63 magnification), demonstrating PAS^+VE^/CD31^+VE^ blood vessels (black arrow) and PAS^+VE^/CD31^−VE^ VM vessels (red arrow). (**c**). Univariate survival analysis according to VM score in LS SCLC. Kaplan–Meier survival analysis for patients dichotomised by VM ratio (percentage of VM vessels/total vessels) into high (*n*=24) and low (*n*=17) VM using a threshold of 11% based on ROC curve analysis. Scale bars, 20 μm, all images are representative.

A tissue microarray (TMA) of LS SCLC (*n*=41) was stained with PAS and anti-human anti-CD31 to investigate the presence of VM (Fig. 2). The clinical characteristics of these patients are summarized in Supplementary Table 1. Figure 2a shows PAS-positive laminin and collagen networks (pink) surrounding tumour cells, identified by enlarged nuclei (blue) and nuclear moulding. PAS^+VE^/CD31^+VE^ endothelial vessels (pink/brown) and PAS^+VE^/CD31^−VE^ VM vessels (pink) were clearly defined (Fig. 2b). VM (Fig. 2b red arrow) and endothelial (Fig. 2b black arrow) vessels were present in all patient specimens.

The VM ratio, expressed as a percentage of total vessels (VM vessels per core/total vessels per core × 100) ranged from 0 to 50% (mean 15.5%, median 12.1%) and receiver operating characteristic analysis for 3-year survival showed an area under the curve of 0.78 (s.e. 0.09, 95% confidence interval 0.61–0.97, *P*=0.008; Supplementary Fig. 2a,b). Over a range of VM ratios, a ‘cutoff' of 11% yielded greatest sensitivity and specificity (Supplementary Fig. 2c). Univariate survival analysis demonstrated that patients with a VM ratio >11% had a reduced 3-year survival (8.3 versus 47.1% alive at 3 years *P*=0.015) and median OS (13.0 versus 23.8 months, log-rank *P*=0.025) compared with those patients with a tumour VM ratio of <11% (Fig. 2c and Supplementary Table 2).

### VM is present in CDX models

**Figure 3:**
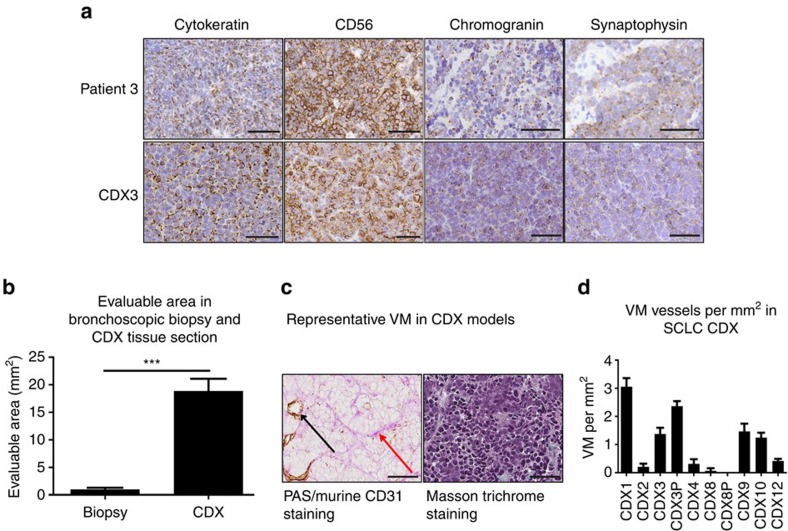
Vasculogenic mimicry in SCLC CDX tumours. (**a**). IHC of CK and SCLC neuroendocrine markers CD56, synaptophysin and chromogranin of a matched patient biopsy and CDX tumour. Scale bar, 50 μm. (**b**) Mean evaluable tumour area (mm^2^) in sections from *n*=24 patient bronchoscopic biopsies and *n*=10 CDX tumours (***P<0.0001, two-tailed *t*-test. (**c**) Left panel depicts PAS^+VE^/CD31^+VE^ endothelial vessels (black arrow) and PAS^+VE^/CD31^−VE^ VM vessels (red arrow) in a representative SCLC CDX tumour. Right panel depicts Masson trichrome staining of the same region demonstrating lack of mouse stromal contamination. Scale bar, 50 μm. (**d**) VM vessels per mm^2^ of tissue in CDX tumours derived from CTCs enriched from SCLC patients (P denotes a CDX tumour derived from a progression blood sample matched to a baseline patient sample, for example, CDX3 and CDX3P are both derived from patient 3 at presentation and progression, respectively). Error bars show s.e.m.

Given the limited tumour availability for this and indeed most studies of SCLC, we sought to confirm that VM occurs in SCLC, exploiting our recently described CDX models^23^. CDX share classic SCLC histological features with patient primary biopsies and cytological specimens (expression of neuroendocrine markers and CKs) and mirror patients' response to chemotherapeutics^23^ (Fig. 3a). In addition, CDX provide significantly more tumour for VM analysis compared with standard bronchoscopic biopsies (18.9 versus 1.0 mm^2^, unpaired *t*-test, *P*<0.0001; Fig. 3b). Using a specific anti-murine anti-CD31 antibody with PAS staining, we identified VM networks in 9/10 CDX models derived from 8 SCLC patients (Fig. 3c). Areas of CDX evaluated for VM were negative for Masson trichrome staining (for example, Fig. 3c right panel) consistent with minimal murine stromal tumour infiltration. VM networks in CDX ranged from 0.00–3.06 VM vessels per mm^2^ (mean 1.05 VM vessels per mm^2^, median 0.83 VM vessels per mm^2^; Fig. 3d).

### Genetics of VM vessel cells in CDX show human tumour origin

**Figure 4:**
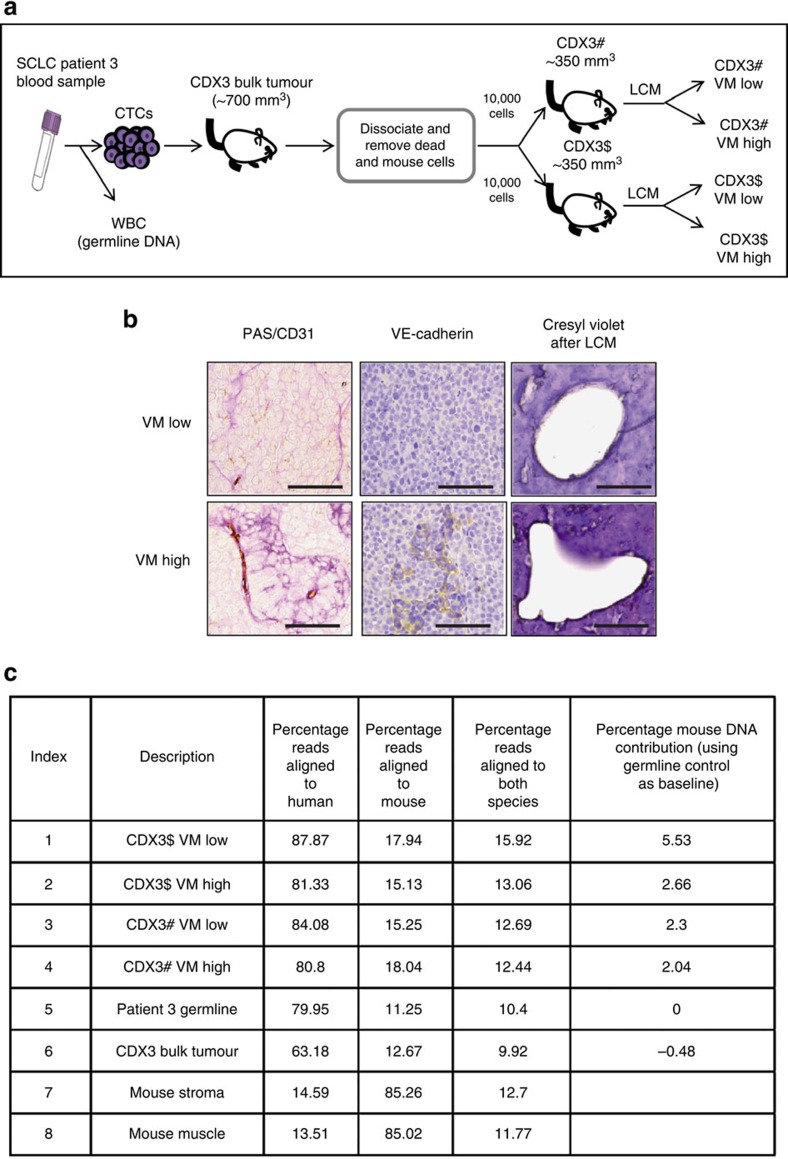
LCM and genomic analysis of VE-cadherin-positive VM vessels in CDX. (**a**) Schematic representation of generation of CDX3 and subsequent samples used for VM analysis, genomic analysis and targeted sequencing of LCM regions. White blood cells (WBC) used to generate germline control DNA. (**b**) IHC staining for PAS/CD31, VE-cadherin and (non DNA damaging) Cresyl Violet with VM-low/-high LCM area from CDX3 tumour. Scale bar, 100 μm. (**c**) Sequencing data from the 8 different samples used for genomic analysis demonstrating minimal mouse cell contamination and predominantly human genomes in VM structures *in vivo*. All reads were aligned to both human and mouse genome separately.

There has been considerable controversy over whether VM vessels are comprised of tumour cells or are an aberrant co-option of healthy somatic cells (reviewed in refs 24, 25, 26). Previous studies in specific tumour types demonstrated the tumour origin of VM vessels either by expression of tumour-specific structures (such as melanosomes in uveal melanoma^7^) or the presence of an introduced mCherry transgene in breast cancer cell lines grown as tumour xenografts^12^. Lacking a marker that would categorically label only SCLC cells, we sought genetic evidence that vessels defined as VM by PAS^+VE^/CD31^−VE^ staining were indeed composed of SCLC cells. We previously reported the generation of CDX3 ref. 23 and here, in two replicate studies (CDX3$ and CDX3#, schematic Fig. 4a), we identified regions of VE-cadherin expressing cells enriched for VM identified by PAS/anti-murine anti-CD31 antibody IHC (Fig. 4b). Laser capture micro-dissection (LCM) was performed on ‘VM-low' (≤20% cells with PAS+/CD31− staining) and ‘VM-high' regions (≥75% cells PAS^+VE^/CD31^−VE^ staining; Fig. 4b). Additional serial sections were stained for VE-cadherin, and for cresyl violet to identify the region for LCM (Fig. 4b). VE-cadherin expression in whole sections of CDX3 tumours was between 5–40% (determined by a Definiens algorithm) and co-localised with regions enriched for VM (defined by percentage of cells that were PAS^+VE^/CD31^−VE^; Fig. 4b). As expected, Masson trichrome staining was evident at the tumour periphery demonstrating murine stroma in these regions (Supplementary Fig. 3), this murine stroma and muscle was subsequently used as controls to confirm our ability to distinguish murine from human sequence reads in the genomic analysis of CDX LCM regions (Fig. 4c and Supplementary Table 3). DNA was extracted from CDX LCM regions and subjected to next-generation sequencing (NGS) with all sequencing reads that aligned to the mouse genome eliminated from the analysis to identify the presence of human-specific sequences (Fig. 4c). For the germline control sample, which does not include any mouse sequences, the unique human reads were identified as being 70% with a similar proportion of unique human sequences also seen for the VM-high, VM-low regions and CDX bulk tumour samples (68–72% Fig. 4c) confirming the human origin of the dissected regions.

**Figure 5:**
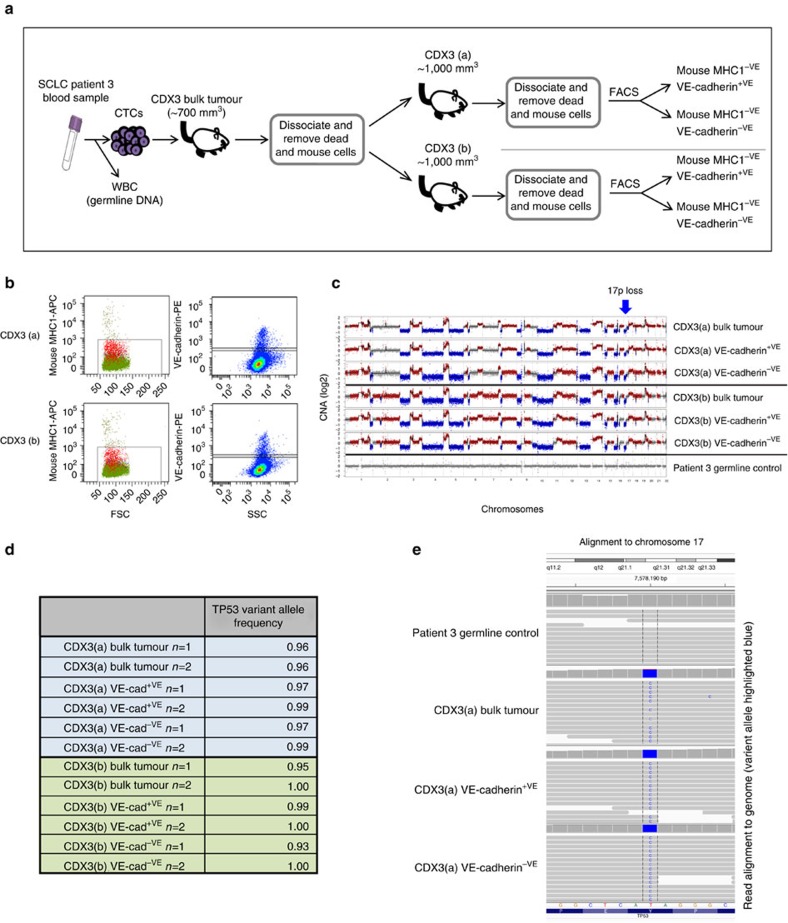
FACS and genomic analysis of VE-cadherin-positive CDX tumour cells. (**a**) Schematic representing generation of CDX3 and subsequent processing of samples for FACS of human (Mouse MHC1^−VE^) subpopulations based on VE-cadherin^+VE^ and VE-cadherin^−VE^ expression. (**b**) Flow Cytometry dot plots showing gating strategies used for cell sorting. Left panels, forward light scatter versus anti-mouse anti-MHC1 staining. Right panels, side light scatter versus anti-human, anti-VE-cadherin staining. Gating strategies set according to relevant controls (see the ‘Methods' section). (**c**) CNA profiles from mice bearing CDX3 tumours (CDX3(a) and CDX3(b)) FACS-sorted mouse MHC^−VE^, bulk tumour/VE-cadherin^+VE^/VE-cadherin^−VE^ and patient 3 germline control (white blood cells). The GC-normalized and mappability corrected read counts (log_2_ scale) were segmented using Hidden Markov Model (HMM), red=copy-number gains, blue=copy-number losses. (**d**) VAF of *TP53* (p.Y220C) mutation in sorted cell subpopulations. (**e**) Read alignment plots from representative samples demonstrate the presence of variant *TP53* allele (p.Y220C) in bulk tumour, VE-cadherin^+VE^ and VE-cadherin^−VE^ fractions, but absent in germline patient control.

Having demonstrated that LCM VM-high regions had enriched expression of VE-cadherin (Fig. 4b), a further two CDX3 tumours (CDX3a and b) were freshly disassociated to single cells, depleted of mouse cells with immunomagnetic beads coupled to anti-murine anti-MHC1 antibody and stained for murine MHC1 and human VE-cadherin. Tumour cell suspensions were sorted by flow cytometry into three subpopulations (in duplicate) of >10,000 cells, total mouse MHC1^−ve^ cells (bulk tumour), mouse MHC1^−VE^/VE-cadherin^−VE^ cells and mouse MHC1^−VE^/VE-cadherin^+VE^ cells (Fig. 5a,b). DNA was extracted from each subpopulation and subjected to low-pass NGS to assess CNA. Minimal evidence of murine read contamination was seen in samples (range 0–2.5% of total reads; Supplementary Table 4), clearly demonstrating samples were of human origin. Although some differences were seen between CDX3a and CDX3b, there was striking similarity across all CNA profiles and all harboured characteristic chromosome 17p loss, a hallmark of SCLC^29^,^30^ (Fig. 5c). Targeted sequencing of the same FACS-sorted subpopulations, including the human VE-cadherin-positive cells, confirmed the previously identified *TP53* mutation (chr17:7578190:T>C (p.Y220C); ref. 22; (Fig. 5d,e)). Taken together the combined data (Figs 4 and 5) strongly support that in SCLC, VE-cadherin-positive VM vessels are tumour cell derived and not the manifestation of co-option of murine host endothelial cells.

### Single-cell CNA confirms VE-cadherin-positive cells are CTCs

We sought to determine whether freshly isolated, single CTCs co-expressing VE-cadherin and CKs had CNA profiles consistent with tumour origin. Blood samples (3 × 10 ml) were collected from an ES SCLC patient consented to a prospective biomarker programme for the investigation of blood-borne biomarkers in lung cancer, conducted at the Christie NHS Foundation Trust, Manchester, UK. The first blood sample underwent EpCam^+VE^/CK^+VE^ CTC enumeration via CellSearch (Janssen Diagnostics, LLC) and contained 156 CTCs per ml, the second, parallel blood sample was analysed using marker-independent High Definition Single Cell Analysis (HD-SCA, see the ‘Methods' section) which identified 501 CTCs per ml. The lower number of CTCs in the CellSearch analysis most likely reflects the presence of EpCam^−VE^ CTCs identified by HD-SCA but not by CellSearch, as we previously observed using another marker-independent CTC platform^31^. Plasma was obtained from the third blood sample and processed for circulating tumour DNA (ctDNA) analysis^32^,^33^.

**Figure 6:**
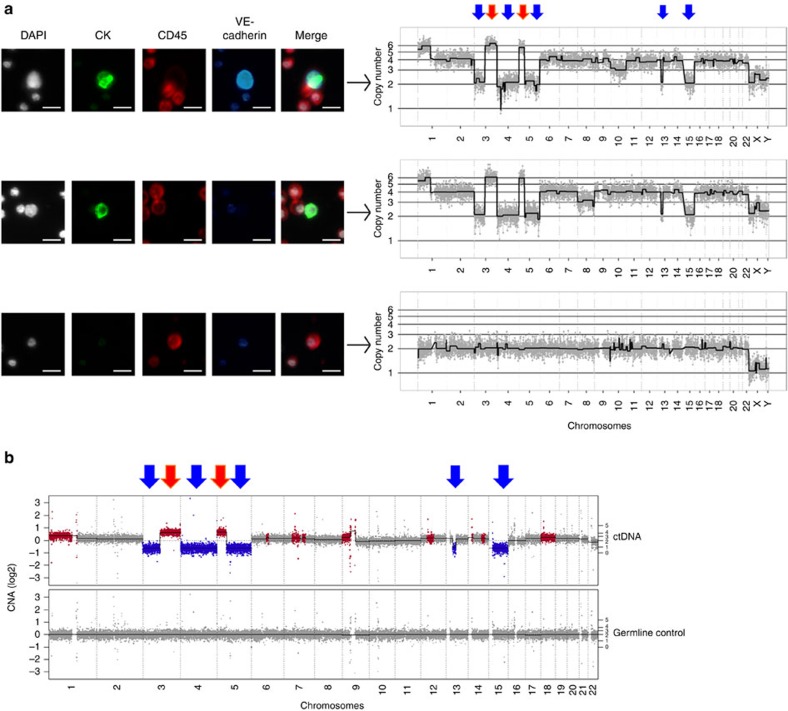
Single-cell CNA analysis of VE-cadherin-negative and -positive CTCs from an ES SCLC patient. (**a**) CTCs identified using the HD-SCA assay: cells were stained with DAPI (white) and antibodies to CKs (green), CD45 (red) and VE-cadherin (blue) and pseudo-coloured. Top panel, DAPI^+VE^/CD45^−VE^/CK^+VE^/VE-cadherin^+VE^ CTC; middle panel, DAPI^+VE^/CD45^−VE^/CK^+VE^/VE-cadherin^−VE^ CTC; bottom panel, DAPI^+VE^/CD45^+VE^/CK^−VE^/VE-cadherin^−VE^ white blood cell (WBC). Arrows link to the respective CNA analysis of the indicated cell, showing loss of 3p, gain of 5p including *TERT* and hemizygous loss of *RB1* on 13 in CTC. Major chromosome losses (blue arrow) and gains (red arrow) highlighted above. The selected WBC in contrast has a characteristic flat CNA profile indicative of healthy somatic cells. Representative images and profiles are shown. Scaled × 10 images from the scanner are shown for CD45, while remaining images were acquired at × 40. Scale bar, 10 μm. (**b**) CNA analysis of matched patient ctDNA: the GC-normalized and mappability corrected read counts (log_2_ scale) were segmented using Hidden Markov Model (HMM), red=copy-number gains, blue=copy-number losses. Major chromosome losses (blue arrow) and gains (red arrow) highlighted above and match pattern identified by single CTC CNA.

Cells identified by HD-SCA assay were stained using immunofluorescent-labelled antibodies to panCKs (green), CD45 (red), DAPI (white) and VE-cadherin (blue; Fig. 6). Using this approach, it was possible to identify both VE-cadherin-positive and -negative, CK-positive CTCs (Fig. 6a). Low-pass genome-wide CNA analysis was performed on 24 isolated single cells (Fig. 6a and Supplementary Fig. 4) and detailed profiles of VE-cadherin-positive and -negative, CK-positive CTCs were compared with a typical white blood cell (Fig. 6a). Notably, loss of chromosome 13q containing *RB1* was observed in both VE-cadherin-positive and -negative CK-positive CTCs. We also evaluated the CNA profile of this patient's ctDNA which was compared with the CNA profiles of the CTCs in their matched blood sample. This comparison showed that the CTC and ctDNA CNA profiles are highly related and typical of SCLC tumours^29^,^30^ (Fig. 6a,b). Although loss of chromosome 17p (harbouring *TP53*, seen in 50–60% of SCLC^30^) was not seen in this patient, targeted sequencing of their ctDNA identified non-synonymous *TP53* and *RB1* mutations (Supplementary Table 5) in keeping with the frequent mutation of these genes in SCLC^34^.

### VE-cadherin plays a functional role in SCLC VM

**Figure 7:**
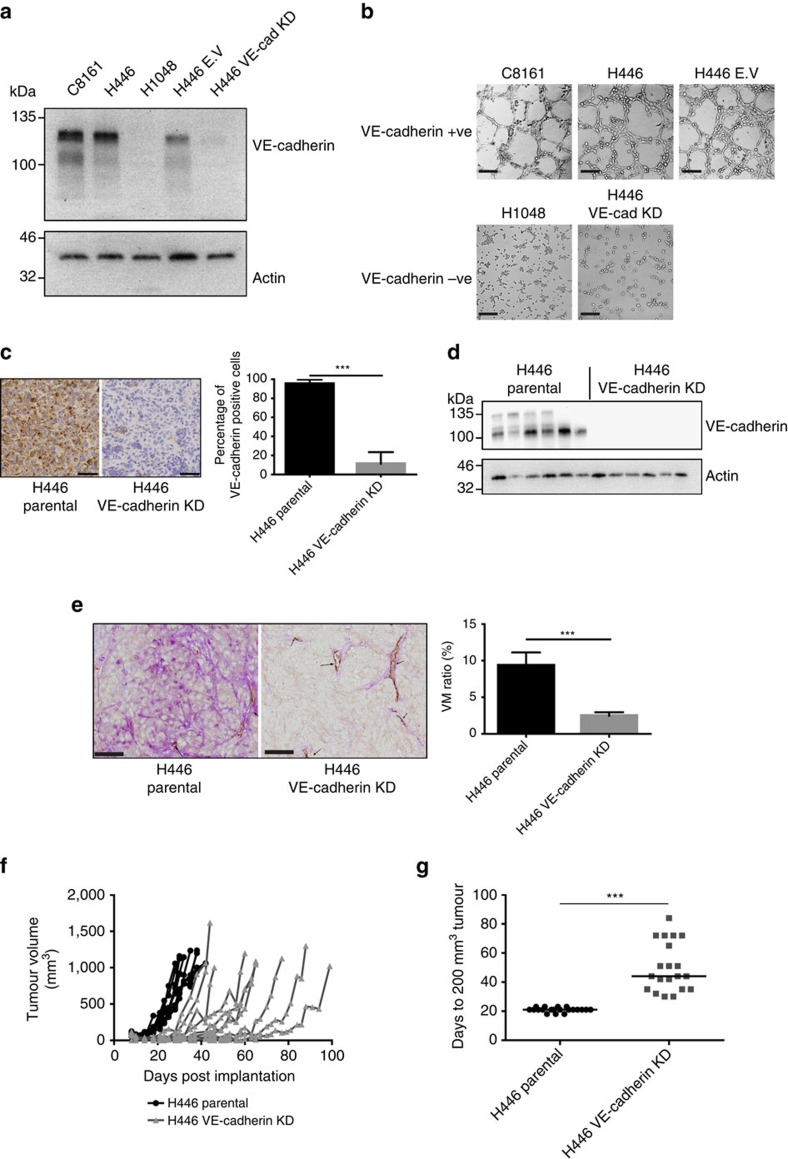
Functional significance of VE-cadherin expression for SCLC VM *in vitro* and *in vivo*. (**a**) Western blot analysis of VE-cadherin expression levels in VM proficient C8161 melanoma cells and H446, H446 non-silencing empty vector controls, H446 VE-cadherin shRNA knockdown and H1048 SCLC cells. (**b**) VM-like network formation on matrigel for VE-cadherin^+VE^ cells (C8161, H446 and H446 E.V) and VE-cadherin^−VE^ cells (H1048 and H446 VE-cadherin KD) lacking network formation. Representative images are shown for *n*>3 experiments. Scale bar, 200 μm. (**c**) Left panel, representative images of anti-human anti-VE-cadherin staining in H446 parental and H446 VE-cadherin KD xenografts. Scale bar, 50 μm. (**c**) Right panel percentage of cells positive for VE-cadherin in H446 and H446 VE-cadherin KD xenografts (***P=0.0001, two-tailed *t*-test), *n*=10 animals per group. (**d**) Western blot analysis VE-cadherin expression in H446 and H446 VE-cadherin KD xenografts (*n*=6 tumours for each group). (**e**) Left panel, representative images of anti-mouse anti-CD31/PAS staining in H446 Parental and H446 VE-cadherin KD tumours. Scale bars, 50 μm. (**e**) Right panel VM ratio in H446 parental (*n*=10) and H446 VE-cadherin KD xenografts (*n*=8; ***P=0.0005, two-tailed Mann–Whitney's test). (**f**) Tumour growth rates in H446 (black) and H446 VE-cadherin KD (grey) tumours (*n*=10 animals per group). (**g**) Days to 200 mm^3^ volume tumours in H446 and H446 VE-cadherin xenografts KD (*n*=20 animals per group, (***P<0.0001, two-tailed *t*-test). Error bars show s.e.m.

Seminal studies of VM in melanoma demonstrated VE-cadherin was essential for VM-associated network formation on matrigel. These VM-like structures have been shown not only to be cellular extensions, but perfusable functional vessels^20^. Gene-expression profiling confirmed that VE-cadherin was upregulated in C8161 uveal melanoma cells competent for VM network formation^13^,^14^,^15^. These studies also demonstrated that depletion of VE-cadherin from C8161 cells, led to abrogation of VM network formation *in vitro* and VM *in vivo*^20^. Subsequent studies validated a functional role of VE-cadherin in glioblastoma and oesophageal cancer VM^34^,^35^. To address the role of VE-cadherin in SCLC VM, a panel of SCLC cell lines were screened for VE-cadherin expression and NCI-H446 (H446) was selected for functional studies (Fig. 7a and Supplementary Fig. 5). H446 cells produced VM-like networks on matrigel which was abolished after VE-cadherin knockdown (VE-cadherin KD) using shRNA; no effect was seen with the non-targeting shRNA transduced cell line control (Fig. 6b). VM-like networks were absent in SCLC cell lines that did not express VE-cadherin, for example, NCI-H1048 (H1048; Fig. 6b). Altogether these data suggest a functional role of VE-cadherin in branching network formation by SCLC cells *in vitro*.

We then asked whether reduced VE-cadherin expression reduced VM in H446 xenografts *in vivo*. VE-cadherin KD cells grown as xenografts maintained a significant reduction in VE-cadherin expression (using the human-specific antibody) throughout the study (91.0 versus 1.5% of tumour cells stain for human VE-cadherin for H446 parental versus H446 VE-cadherin KD, *P*<0.0001; Fig. 7c,d, Supplementary Fig. 6). The reduction of tumour VE-cadherin expression correlated with reduction in VM vessels (9.36 versus 2.49 VM scores for parental H446 versus H446 VE-cadherin KD *P*=0.0005; Fig. 7e). In addition, we demonstrated that reduction in VM vessels impacted tumour growth dynamics; on average VE-cadherin KD H446 tumours had a 22.3-day increase in lag time to reach a tumour volume of 200 mm^3^ (H446, 21.0 days versus H446 VE-cadherin KD, 43.3 days, *P*<0.0001; Fig. 7f,g).

### VE-cadherin KD impairs cisplatin delivery into tumours

**Figure 8:**
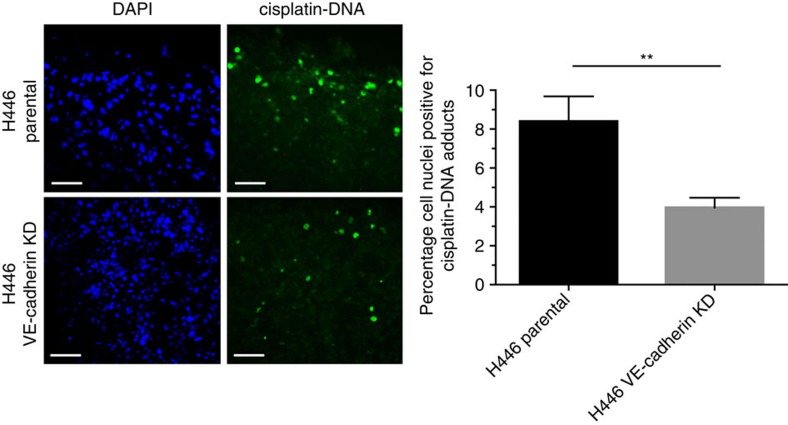
Cisplatin delivery in H446 and H446 VE-cadherin KD xenografts. Left panel, immunofluorescence assay for cisplatin–DNA adduct formation in nuclei of H446 and H446 VE-cadherin KD xenografts 1 h after dosing. Scale bar, 50 μm. Right panel, percentage of nuclei positive for cisplatin–DNA adducts in H446 and H446 VE-cadherin KD xenografts (*n*=15 tumours per group (**P=0.0052, two-tailed Mann–Whitney's test)). Error bars show s.e.m.

We hypothesized that the presence of VM might increase the delivery of chemotherapy into tumours. To test this hypothesis, we assessed the intra-tumoural delivery of cisplatin using an antibody to cisplatin–DNA adducts for tumours with reduced VM due to VE-cadherin KD (Fig. 7c). Cisplatin–DNA adduct formation *in vivo* in VE-cadherin KD tumours versus parental controls was decreased twofold, 1 h following a single dose of cisplatin (i.p.), in size-matched tumours (Fig. 8, left panel); 8.4 versus 4.0% of nuclei stained positively for cisplatin adducts in H446 parental versus H446 VE-cadherin KD xenografts (*P*=0.0052; Fig. 8, right panel). We then asked whether reduction in cisplatin uptake by VE-cadherin KD tumours reflected intrinsic differences in intracellular drug influx/efflux and subsequent response to cisplatin. H446 parental and H446 VE-cadherin KD cells were assessed *in vitro* for sensitivity to cisplatin. Knockdown of VE-cadherin led to a significant increase in sensitivity to cisplatin *in vitro* (IC_50_ 12.29 versus 3.23 nM, for H446 parental versus H446 VE-cadherin KD, *P*<0.0001; Supplementary Fig. 7a,b), consistent with the reduced cisplatin uptake by tumours (Fig. 8) due to impaired drug perfusion rather than a cell intrinsic increase in cisplatin resistance.

### VE-cadherin knockdown increases chemosensitivity *in vivo*

**Figure 9:**
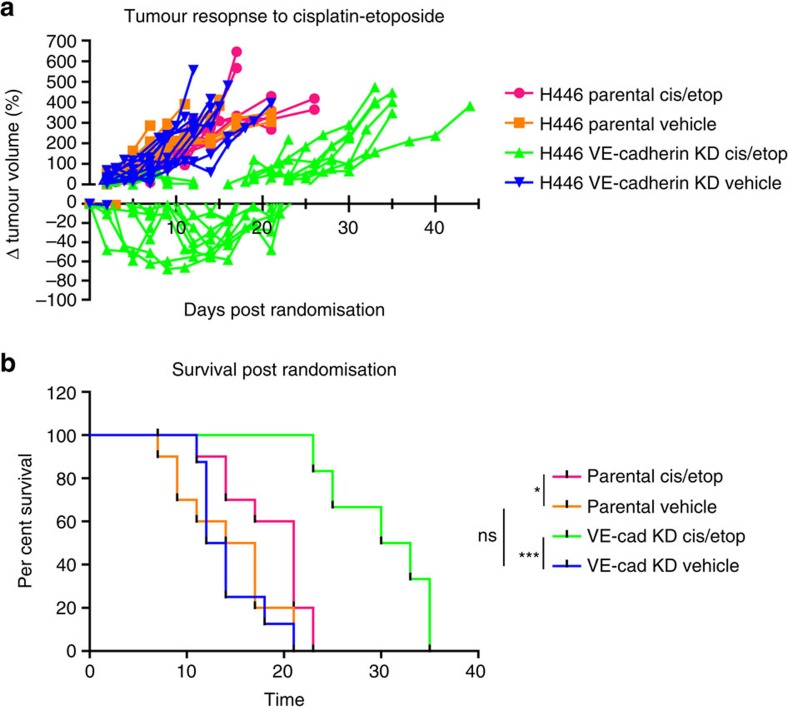
Efficacy of cisplatin-etoposide treatment in H446 and H446 VE-cadherin KD xenografts. (**a**) Tumour response measured post randomization as a percentage tumour volume change relative to size at randomization (*n*=10 tumour-bearing animals per group). (**b**) Univariate survival analysis following cisplatin/etoposide treatment in H446 parental and H446 VE-cadherin KD xenografts. Kaplan–Meier survival analysis for tumours treated with vehicle or cisplatin/etoposide (*n*=10 animals per group (ns=non-significant, *P=0.03, ***P=0.0003, Mantel–Cox test)).

We next sought to investigate the impact of VE-cadherin knockdown with attendant reduced VM on tumour responses *in vivo* to the standard of care chemotherapy doublet, cisplatin and etoposide. Although H446 VE-cadherin KD cells were more sensitive to cisplatin *in vitro* (Supplementary Fig. 7), knockdown of VE-cadherin did not affect sensitivity of cells to etoposide *in vitro* (IC_50_ 2.75 versus, 3.00 nM for H446 parental H446 versus VE-cadherin KD, *P*=0.600; Supplementary Fig. 8). Mice were implanted with H446 parental or H446 VE-cadherin KD cells and tumours were allowed to grow to 200 mm^3^ before being randomised and dosed with a single dose of 5 mg per kg cisplatin and 8 mg per kg etoposide on 3 consecutive days (i.p.), or equivalent vehicle. H446 parental tumours had a minimal response to dosing with cisplatin/etoposide when compared with vehicle-treated animals (Fig. 9a). In contrast, a reduction in tumour volume (percentage of tumour volume change relative to size at randomization) occurred with H446 VE-cadherin KD tumours following cisplatin/etoposide treatment (mean −43.4% reduction, median −47.9% reduction, range −9.4% to −68.1% reduction; Fig. 9a). Treatment of H446 parental xenografts with cisplatin/etoposide led to a 5.50-day median increase in survival compared with vehicle-treated mice (*P*=0.028; Fig. 9b). Treatment of H446 VE-cadherin KD with cisplatin/etoposide resulted in an 18.50-day median increase in survival (*P*=0.0003) compared with vehicle-treated mice (Fig. 9b). No significant difference in survival was seen between vehicle-treated H446 parental and H446 VE-cadherin KD tumours following randomization (*P*=0.84; Fig. 9b).

## Discussion

VM has been described in several human cancers and associated with pluripotent ‘stem' cell marker expression, aggressive disease and poor patient outcomes^13^ with emerging evidence of VM involvement with tumour dissemination and metastasis^12^. This association with aggressive disease has led to efforts to seek VM-targeted therapies^36^. We posited that if VM enhanced haematogenous dissemination, cells with a VM phenotype would be present in the circulation of cancer patients. Here, we report for the first time, that a rare subset of CTCs in SCLC express VE-cadherin, an established marker of VM (Figs 1 and 6). We infer that this subpopulation of VM CTCs may support VM network capability in secondary tumours.

We show that VM vessels are present in SCLC patient tumour specimens and that a high VM ratio correlates with poor prognosis in LS disease (Fig. 2, Supplementary Fig. 2 and Supplementary Table 2), observing that 21 of the 29 LS patients with survival <3 years have a high VM ratio. This result is consistent with the hypothesis that VM acts through aggressive tumour ‘stem-like' phenotypes, and tumour plasticity^6^,^37^, leading to worse clinical outcomes. Furthermore, 7 out of the 10 LS patients who survived >5 years, (17% of the total cohort studied) had low VM ratios. As the reported 5-year survival rate for LS disease is 12−15% (refs 38, 39, 40), VM status may be a novel prognostic biomarker. A prospective study will be essential to confirm the negative impact of VM on SCLC prognosis and tractable surrogate models are needed to interrogate the molecular regulation of VM and investigate further its impact on SCLC biology and response to treatment.

Obtaining tumour specimens for research is challenging in SCLC where <5% patients undergo tumour resection^38^. The development of CDX affords a new opportunity to study SCLC biology. We confirm here that ample CDX tissue, relevant to the donor patient, can be generated for VM analysis, not possible using bronchoscopic biopsies (Fig. 3a,b). CDX models will also permit putative VM-targeted drug testing, for example, of therapies that inhibit VE-cadherin signalling^41^,^42^. Mutations in Notch family genes have been recently reported in 25% SCLC^29^ and whilst these signalling networks are complex, it is notable that inhibitors of Notch signalling are reported to preferentially target ‘stem-like' VM tumour cells^43^,^44^,^45^.

Whether VM vessels are derived from *bona fide* tumour cells that have trans-differentiated, or instead represent a cell subpopulation of endothelial lineage, has remained somewhat controversial regarding cancer types where there is an absence of specific tumour markers. Here, we present the first genomic evidence in CDX that VE-cadherin-positive VM vessels are not composed of mouse endothelial cells, but rather they are human SCLC cells (Figs 4 and 5 and Supplementary Tables 3 and 4). Although there is a VE-cadherin expressing VM CTC subpopulation in the vast majority of patents, the actual numbers of VM CTCs detected are low (Fig. 1b). Low numbers of VE-cadherin expressing CTCs were also seen for the patient chosen for HD-SCA analysis with only a single VE-cadherin expressing CTC detected from the 18 CTCs examined (Fig. 6 and Supplementary Table 5). Nevertheless, the single VE-cadherin expressing CTC exhibited essentially the same copy-number gains and losses observed in the VE-cadherin CTCs and the corresponding patient ctDNA (Fig. 6, Supplementary Fig. 4 and Supplementary Table 5) clearly indicating the common SCLC origin of all CTCs examined.

We next addressed the pivotal question of whether VM is of functional significance in SCLC using VE-cadherin KD H446 cells (Fig. 7). Knockdown of VE-cadherin significantly reduced VM and altered tumour growth kinetics. We speculate that the increased latency for tumour growth observed with this reduction in tumour VM may result from an enhanced requirement for host angiogenesis to occur to supply nutrient and oxygen for tumour growth.

Reduction of tumour VM also affected how SCLC xenografts responded to chemotherapy, consistent with the concepts linking ‘stem cell' like plasticity, VM and drug resistance^46^,^47^. Despite the observed increased cisplatin delivery to SCLC xenograft tumours with unperturbed VM (Fig. 8), there was decreased response to cisplatin and etoposide (Fig. 9) compared with that in the corresponding xenografts with reduced VE-cadherin expression and reduced VM. As this xenograft model system consists of a parental tumour where all cells express VE-cadherin and a VE-cadherin shRNA knockdown counterpart where all detectable VE-cadherin expression was lost (Fig. 7d), we were unable to assess whether there would be an enrichment of chemo-resistant VE-cadherin-positive cells following chemotherapy. It does, however, provide an initial proof of concept that VE-cadherin-positive cells competent for VM may be one of many mechanisms of cisplatin resistance in SCLC. Consistent with this hypothesis in another neuroendocrine tumour, Merkel cell carcinoma of the skin, VM-rich areas of tumour were refractory to platinum-etoposide chemotherapy^21^. Taken together, the data comparing SCLC cells with and without VE-cadherin knockdown demonstrate that VE-cadherin plays a functional role in SCLC VM, as seen in melanoma and glioblastoma^20^,^35^. Overall, our data suggest that even though rare, VM competent SCLC cells may be able to promote outgrowth of chemo-resistant clones preceding treatment failure. Future studies will focus on the modulation of VE-cadherin expression in CDX models and lineage tracing of VE-cadherin expressing cells *in vivo* before and after therapy. Furthermore, a prospective study exploring VE-cadherin expression in biopsies or (more readily available) CTCs at baseline and again at disease progression with chemotherapy resistance will highlight to what extent VE-cadherin-positive tumour cells are enriched during development of chemoresistance in the clinic. With a recent resurgence in interest in VM^26^, further studies are warranted to determine precisely how VE-cadherin signalling regulates VM in SCLC and how VE-cadherin signalling could be exploited as a therapeutic target in this most recalcitrant cancer.

## Methods

### Patient samples

Patients with histological or cytological confirmation of chemotherapy-naive SCLC attending the University Hospital of South Manchester or Christie NHS Foundation Trust were recruited to our broader spectrum of SCLC research (European Union CHEMORES FP6 Contract number LSHC-CT-2007-037665) according to ethically approved protocols (NHS Northwest 9 Research Ethical Committee). VM was evaluated in a TMA from 41 LS SCLC patients^48^ and in blood samples from 38 LS or ES patients for enrichment of CTCs by ISET filtration (method described in ref. 49; bronchoscopic biopsies were also obtained from 24 of the ES patients for whom CTCs were analysed. A separate cohort of 9 ES patients provided blood samples that led to the derivation of 11 CDX^23^ that were used to assess the prevalence of VM vessels and for CDX3L, CNA profiles of CDX tumour regions with high and low VM. Finally, CTCs from an additional ES SCLC patient were analysed using the EPIC platform and subsequent isolation of single CTCs for CNA profiling.

### Immunofluorescent analysis of ISET-filtered CTCs

Tumour cells in 7.5 ml blood samples were immobilized on ISET filters (Metagenex) according to the manufacturer's recommendations^22^. Cells on ISET filters were permeabilised with phosphate-buffered saline (PBS) with 0.2% Triton X-100 (PBST), blocked with 10% goat serum (Dako, X090710) in PBST before staining for rabbit anti-human CD45 (1:1,000, Abcam, AB10559) and mouse anti- human VE-cadherin (1:200, eBioscience, 14-1449-82). Cells were washed twice with PBST then counterstained with goat anti-rabbit DY550 (1:500 Thermo Fisher Scientific, Wilmington, USA, catalogue number 84541) and goat anti-mouse Alexa-Fluor 647 (1:500 Invitrogen, catalogue number A-21235) washed twice again in PBST then stained with anti-human pan-cytokeratin-FITC (1:100 Sigma, clone C-11) Filters were washed twice in PBST, incubated with DAPI (0.5 μg per ml, Invitrogen D3571, 80 μl, 5 min), washed twice in PBST then double-distilled water before mounting on a glass slide with ProLong Gold anti-fade (Invitrogen P36934). Slides were left overnight at room temperature (RT) to dry before imaging with a MIRAX automated slide scanner (3DHistech, Budapest, Hungary). Images were reviewed with 3DHistech panoramic viewing software (version 1.15.1.10). CTCs were defined as CK^+VE^/CD45^−VE^. VM score was calculated from a 4 ml blood sample as VE-cadherin^+VE^/CK^+VE^/CD45^−VE^ CTCs/total number of CTCs per ml blood as percentage.

### VM detection and scoring of TMA and CDX samples

Formalin-fixed, paraffin-embedded sections (4 μm) of TMA and CDX were stained by immunohistochemistry (IHC) for pan-CK; pan-CK antibody to CKs 1–8,10, 13–16 and 19, mouse AE1/AE3, M3515, 1:60, Dako), CD56 (mouse, 1B6, NCL-CD56-aB6, 1:100, Novocastra), synaptophysin (mouse, 27G12, NCL-L-SYNAP-299, 1:200, Novocastra), anti-human VE-cadherin (mouse, Clone16B1, 1:50, eBioscience) anti-human CD31 (mouse,Clone JC70A, 1:100, Dako/M0823) and anti-mouse CD31 (rabbit, a kind gift from N.R. Smith, AstraZeneca, 1:600). For all antibodies,except Chromograin A anti-Mouse CD31, IHC assays were carried out on a LEICA Bond Max platform, using standard protocol F^50^. Negative IgG controls were performed for each tissue section and treated identically to experimental slides.

Chromogranin A (mouse, LK2H10+PHE5, MP-010-CM1, 1:600, Menapath) Antibody incubations and detection were carried out at RT on a Menarini IntelliPATH FLX (A. Menarini Diagnostics) using Menarini's reagent buffer and detection kits.

Antigen retrieval was performed in a pressure cooker using access super retrieval fluid (MP-606-PG1), which was incubated for 10 min with protease (MP-960-K15) on IntelliPATH. For the CD31/PAS double-staining, sections underwent antigen retrieval in DakoCytomation Target Retrieval Solution Citrate pH6 (S2369) at 110 °C for 5 min before being stained by IHC for CD31 on a Biogenex i6000 Autostainer. Stained sections were viewed with a light microscope at a magnification of × 40 and analysed by two independent scorers (R.M. and F.T.) without knowledge of demographic or outcome data. Suitable digital images were captured using MIRAX brightfield digital slide scanner (3DHistech) with Panoramic Viewer software. CD31^+VE^ Vessels/lined spaces were defined as endothelium-dependent vessels. VM channels were defined as PAS^+VE^/CD31^−VE^ channels enclosed by SCLC cells (the absence of endothelial cells confirmed by hematoxylin-eosin staining). The entire scorable area of each section was used to calculate the total number of VM channels and endothelium-dependent vessels. The percentage of VM structures in relation to the total number of vessels in each of the TMA cores or CDX sections was calculated as the ratio of VM vessels (PAS^+VE^/CD31^−VE^)/total vessels (PAS^+VE^/CD31^+VE^ plus PAS^+VE^/CD31^−VE^) per section expressed as a percentage^10^.

VE-cadherin expression was scored using Definiens Developer XD (version 2.0.4) and Tissue Studio Portal (version 4.2 ), (Definiens AG, Munich, Germany). Regions of interest were identified through machine learning across several sections using Definiens Tissue Studio^51^. Nuclei were detected within these regions of interest and classified as positive or negative for staining based on a threshold set as a constant across all tissue sections.

IHC for cisplatin–DNA adducts in xenografts containing H446 or H446 VE-cadherin KD cells treated with cisplatin or vehicle was performed essentially as described elsewhere^51^, except with the following modifications: briefly, samples were fixed in methanol (10 min, 4 °C) and rehydrated in PBS (10 min; 25 °C). Cellular RNA was digested by RNase treatment (RNase A, 200 mg per ml in PBS; 100 ml per slide; Roche; 1 h; 37 °C) in a humidified chamber. Cellular proteins were digested with pre-warmed ficin (Invitrogen REF 003007, ready to use) for 10 min at 37 °C followed by pre-warmed proteinase K (Novocastra RE7160CE; dilute 1 drop per ml PBS) for 10 min at 37 °C. After blocking with Avidin/biotin block (Vector lab, SP2001) for 30 min at 37 °C and PBS wash, slides were incubated with primary antibody (1:1,000 in PBS with 1% casein, Millipore rat Ab against cisplatin–DNA, clone ICR4, Cat. # MABE416) for 2 h at 37 °C. Following PBS wash slides were incubated with FITC-labelled secondary antibody (1:400, anti-rat, diluted in Ab diluent, 712-066-153, Jackson ImmunoResearch) for 1 h at 37 °C and further incubated with Alexa-Fluor 488-labelled anti-FITC antibody (1:1,000, diluted in Ab diluent, Invitrogen, catalogue number S11223) for 30 min at 37 °C and counterstained with ProLong Gold Antifade Mountant with DAPI (catalogue number P36935, Thermo Fisher Scientific). Visualization and quantitation of antibody- and DNA-derived fluorescence was performed via low-light microscopy. Slides were blinded and the percentage of Cisplatin–DNA-positive cells was calculated from manual scoring of 10 random fields at × 40 magnification by two independent operators.

### Statistical analysis

Statistical analysis was performed using SPSS version 20 (SPSS, Chicago, IL, USA). Fisher's exact *t*-test was used to evaluate for association between the TMA VM score and prognostic factors (hemoglobin <9 g per l, white blood cell >10 × 10^9^ per l, platelets <150 × 10^9^ per l, Na <135 mmol per l and LDH >550 IU per l^11^). Univariate survival analysis was performed using Kaplan–Meier analysis and differences between survival distributions were evaluated by Log-rank test. A *P* value of <0.05 was considered statistically significant. Survival times were calculated as time elapsed between biopsy date and date of death or last follow-up. Patients alive at last follow-up were censored.

To determine a threshold for the TMA VM score with greatest sensitivity and specificity for prediction of 3-year survival, receiver operating characteristic curves were analysed to calculate area under the curves and to determine the sensitivity and specificity of the prediction at multiple thresholds.

For *in vivo* studies, to detect a fourfold effect size with 80% power and a significance level of 0.05, a group size of *n*=9 was required. All studies used a minimum of 10 animals per group.

### Identification of CDX tumour regions with high and low VM

CDX3 was derived from a male patient with ES SCLC^23^. CDX3 was passaged three times and at a tumour volume of 664 mm^3^, the tumour disaggregated with removal of dead and murine cell contaminants (using magnetic beads) and 10,000 SCLC cells were re-implanted into 2 female NSG mice. Mice bearing the resultant CDX3# and CDX3$ tumours (∼300–400 mm^3^) were killed and tumours were formalin-fixed. Three consecutive sections were stained by IHC for VM (CD31/PAS), synaptophysin and VE-cadherin (as above), to identify regions of high and low prevalence of VM for LCM of the parallel sections and copy-number alteration (CNA) analysis. With VM-high and -low regions thus identified, a further eight consecutive tissue sections were cut and directly mounted on Frame slides PET membrane (Leica Microsystems GmbH, Wetzlar, Germany) for LCM.

### LCM of CDX VM-high and -low regions

Slides containing VM-high and -low regions in were stained with 4% Alcoholic Cresyl violet and 0.5% alcoholic Eosin (to maintain DNA quality). Separate regions of CDX3# and CDX3$ were identified according to their VM-high (≥75% area consisting of VM contributing cells) and VM-low phenotype (≤20% area consisting of VM contributing cells) by PAS/CD31 staining. To obtain sufficient cells to asses CNA in both VM-high and -low regions, ∼150 cells in each of the eight sections (that also stained for synpatophysin by IHC in a consecutive section) were laser captured, micro-dissected from the Frame slides (LCM 6000 B Leica Microsystem).

### FACS of CDX tumours based on VE-cadherin expression

CDX3 tumours were dissociated to single cells with Miltenyi Biotecs tumour dissociation kit according to manufacturer's recommendations on a gentleMACS dissociator^52^. Single cells underwent immunomagnetic depletion of mouse cells and dead cells with a Dead cell removal microbead set (Mitenyi Biotec), anti-mouse anti-MHC1 antibody (eBioscience clone, 34-1-2s) and anti-mouse anti-IgG2a+b microbeads over a LS column in a MidiMACS Seperator (Miltenyi Biotec). Depleted cells were stained with anti-human anti-VE-cadherin-biotin (mouse, 1:200 eBioscience, clone 16B1), streptavidin-PE (1:500, catalogue number 12-4317-87, eBioscience) and anti-mouse anti-MHC1-APC (mouse, 1:200, clone 34-1-2s, eBioscience), before undergoing sorting by FACS to populations of >10,000 cells per group (in duplicate) on a FACSAria III cytometer (BD Biosciences, Heidelberg, Germany). Antibody combinations with BD CompBeads anti-mouse anti-IgG (catalogue number 552843, BD Biosciences) according to manufacturer's recommendations were performed for fluorescence emission spectral compensation and cytometry gating strategies were set according to fluorescence minus one- stained CDX3 cells.

### CNA analysis of CDX-dissected regions

DNA was extracted and amplified from a total of ∼1,200 LCM cells (150 cells in 8 sections combined) from each VM-high and VM-low region of CDX3# and CDX3$ with PicoPLEX for whole-genome amplification (WGA; WGA Kit Rubicon Genomics). WGA products were purified (Agencourt AMPure XP, Beckman Coulter) and DNA concentrations calculated (Nanodrop1000, Thermo Fisher Scientific) and Qubit (Life Technologies). WGA samples were assessed for quality control (Ampli1 QC kit, Silicon Biosystems, Bologna, Italy). In parallel, DNA from passage 1, bulk CDX3 tumour (containing a mixture of VM-high and VM-low regions) and matched patient blood to obtain germline was extracted (DNA Mini Kit, Qiagen, Valencia, CA, USA) and used as a positive internal control for downstream analysis. Samples passed QC if the correct-length amplicons were visualized on a 2% agarose gel. For CNA analysis, the DNA library was prepared from 50 ng of amplified DNA (NEBNext Ultra DNA Library Prep Kit, Illumina, New England Biolabs, Ipswich, UK). Each library was quantified (KAPA qPCR Bioanalyzer, KAPA Biosystems, MA, USA) and equimolar amounts were pooled and sequenced on a MiSeq Illumina platform (150 b.p. pair end).

### Circulating cell-free DNA preparation and quantification

Blood samples collected in EDTA (ethylenediaminetetraacetic acid) Vacutainer tubes (BD Biosciences) were used for extraction of germline DNA and blood samples collected using Cell-Free DNA BCT tubes (Streck, Omaha, NE) were used for extraction of circulating cfDNA from a patient with ES SCLC. Plasma from Cell-Free DNA BCT blood samples, was separated from whole blood by two sequential centrifugations (each 2,000*g*, 10 min) followed by upper phase plasma removal and stored at −80 C in 2 ml aliquots^32^. cfDNA was isolated from 4 ml of double-spun plasma using the QIAamp Circulating Nucleic Acid Kit (Qiagen, Hilden, Germany) as per manufacturer's instructions. Following isolation, cfDNA yield was quantified using the TaqMan RNase P Detection Kit (Life Technologies) as per manufacturer's instructions^32^. Germline DNA was isolated from thawed EDTA whole blood using QIAmp Blood Mini Kit (Qiagen) as per manufacturer's instructions.

### NGS sequencing of FACS-sorted CDX cells and patient ctDNA

Libraries for whole-genome sequencing of cfDNA and corresponding germline DNA from the patient with ES SCLC and FACS-sorted CDX3 tumour samples were carried out using the Accel-NGS 2S Plus DNA Library Kit and indexing of the libraries using NEBNext Multiplex oligos for Illumina (New England Biolabs) using a 25 ng DNA input. Libraries were quantified by qPCR using the Library Quantification Kit for Illumina sequencing platforms (KAPA Biosystems, Boston, USA) as per manufacturer's instructions on the LightCycler 96 System (Roche). Libraries were pooled in equimolar proportions and paired-end sequencing (300 cycles) was performed on the Illumina MiSeq (Illumina) benchtop sequencer using the MiSeq Reagent Kit v2 (Illumina).

### Targeted NGS analysis

Targeted NGS of 110 SCLC-associated genes for cfDNA and corresponding germline DNA from the patient with ES SCLC was carried out using Agilent SureSelectXT essentially as described previously^33^. In brief, we enriched the 110 SCLC-associated genes using SureSelectXT Custom DNA Kit (Agilent, Santa Clara, CA, USA) following the manufacturer's recommendations. The enriched DNA libraries were re-amplified using the KAPA HiFi PCR Kits and Illumina sequencing primers for 30 cycles. Paired-end sequencing was performed for these enriched libraries on the Illumina NextSeq 500 (Illumina) benchtop sequencer with the NextSeq 500/550 v2 kits.

Targeted NGS of the 10 SCLC-associated genes (*KRAS*, *TP53*, *RB1*, *PIK3CA*, *PTEN*, *BRAF*, *NRAS*, *ALK*, *NOTCH1*, *EGFR*) for the CDX3 tumour DNA samples was carried out using custom xGen Lockdown Probes (Integrated DNA technologies). In brief, 500 ng adaptor-ligated DNA libraries from 6 FACS-sorted CDX3 tumour samples were pooled together and combined with 5 μg of Cot-I DNA (Invitrogen) and 1 nmol each of blocking oligonucleotides (TS-p5 and TS-p7). The mixture was lyophilized and then re-suspended in 1.8 μl water, 8.5 μl 2 × xGen hybridization buffer and 2.7 μl xGen hybridization buffer enhancer. The mixture was heated to 95 °C for 10 min. Subsequently, 3 pmol of cuxtom xGen lockdown probes were added and allowed to hybridize for 4 h at 65 °C. After hybridization, M-270 streptavidin beads (Life Technologies) were added and washes were performed according to the manufacturer's recommendations. The enriched DNA libraries were re-amplified using the KAPA HiFi PCR Kits and Illumina sequencing primers for 30 cycles. The amplified enriched libraries were then sequenced on the Illumina MiSeq (Illumina) benchtop sequencer using MiSeq Reagent Kit v2 (Illumina).

### Copy-number and mutation analysis of the CDX samples

*Alignment and processing of sequencing reads*. Sequencing reads were aligned to the human reference genome GRCh37/hg19 using the Burrow–Wheeler aligner (version 0.7.7) (ref. 53) with default parameters. To remove potential contaminating mouse DNA, we aligned the sequencing reads to mouse reference GRCm38/mm10 independently and discarded reads that cross-aligned to both human and mouse genomes.

*Copy-number analysis*. The Bioconductor package HMMcopy (version 1.8.0) (refs 54, 55) was used to determine copy-number variations. Briefly, the genome was divided into 150-kb windows. Reads in each window were normalized by GC-content and mappability. Then a Hidden Markov Model-based approach was used to segment the data into regions of similar copy-number profile and to predict a CNA event (that is, 0, 1, 2, 3, 4 or ≥5 copies of chromosome) for each segment.

*Somatic mutation calling of CDX samples (IDT 10 gene enrichment)*. After removing mouse reads, Picard (version 1.96, http://picard.sourceforge.net) was used to mark duplicated reads. The alignments were sorted and indexed using SAMtools (version 0.1.19) (ref. 56). Local re-alignment around known INDELs and base quality recalibration were performed using Genome Analysis Toolkit (GATK, version 3.1.1) (ref. 57). The recalibrated alignments of the tumour and matched germline control samples were analysed in MuTect (version 1.1.5) (ref. 58) to detect somatic single-nucleotide variants (SNVs). MuTect was run in the High-Confidence mode with default settings except that a maximum of 5 variant alleles was allowed in the germline control (--max_alt_alleles_in_normal_count=5). ANNOVAR (version 2015 Mar 22) (ref. 59) was used to annotate the call sets.

*Somatic mutation calling of cfDNA and Germline samples (Agilent 110 gene enrichment)*. The aligned reads in SAM format were converted to BAM format and sorted using Picard tools (version 1.96, http://picard.sourceforge.net). Local re-alignment around known INDELs and base quality recalibration were performed using GATK (version 3.1.1) on the sorted and marked duplicates BAM files. MuTect (version 1.1.5) was used to detect somatic SNVs in the matched tumour-germline control samples. An SNV was included in the final call set (Supplementary Table 5) if it fulfills the following criteria: (1) variant allele frequency (VAF) in tumour sample ≥0.025, (2) VAF in tumour is at least 5 times higher than VAF in the germline control and (3) it is covered by least 200 reads in both tumour and germline samples. Finally, the SNVs were annotated using ANNOVAR (version 2015 Mar 22).

### HD-SCA isolation of single VE-cadherin-positive CTCs

Peripheral blood samples were collected, stained and imaged using the HD-SCA assay^1^. On arrival of blood samples, white blood cell counts were measured using a M-Series Hematology Analyzer (Medonic, Stockholm, Sweden) to determine the blood volume required to plate 3 million cells in a monolayer onto adhesion slides (Marienfeld, Lauda, Germany). The erythrocyte population was lysed while rocking the sample for 5 min at RT in NH_4_Cl solution. The lysed blood was centrifuged at 800*g* and the nucleated cells were re-suspended in PBS and incubated for 40 min at 37 °C on the adhesion slides. To block unbound sites, slides were incubated with 7% BSA for 5 min at RT. The BSA was discarded and a coverslip was attached to dried slides before freezing at −80 °C for long-term storage.

Before staining, slides were thawed and cells were fixed with 2% PFA for 20 min at RT, washed with PBS and permeabilized with 100% ice-cold methanol for 5 min at RT. After washing with PBS twice, slides were blocked with 10% goat serum at 37 °C for 20 min. A cocktail of antibodies containing 140 μg per ml pan-CK (Sigma, St Louis, MO, USA), 0.2 μg per ml CK 19 (DAKO, Carpinteria, CA, USA), 10 μg per ml CK 7 (Millipore Billerica, MA, USA), 1.6 μg per ml Alexa-Fluor 647-conjugated CD45 (Serotec, Raleigh, NC, USA) and 92 μg per ml VE-cadherin (Cell Signalling Technology, Danvers, MA, USA) in 10% goat serum was allowed to bind at 37 °C for 40 min. Residual non-binding antibody was removed by washing with PBS twice, and a mix of 100 ng per ml DAPI, 4 μg per ml Alexa-Fluor 555 goat anti-mouse (Thermo Fisher Scientific, Waltham, MA, USA) and 8 μg per ml Alexa-Fluor 488 goat anti-rabbit (Thermo Fisher Scientific) were incubated at 37 °C for 20 min. After another round of washing, slides were dipped in dH_2_O and mounting media was added before cover slips were attached using nail polish. Slides were imaged by a custom made fluorescent scanning microscope. Candidate cells were computationally identified and presented to pathology trained personnel. VE-cadherin-positive or -negative SCLC cells were classified and re-located for single-cell genomics studies^60^.

### High-content single-cell selection and CNA of CTCs

Cells were subsequently isolated using a micropipette and subjected to WGA (WGA4, Sigma-Aldrich, St Louis) and low-depth Illumina sequencing yielding ∼1 × 10^6^ uniquely mapping reads. Copy-number profiles were generated by grouping the reads into 4,973 bioinformatically derived bins (∼0.5 Mbp) containing equal amounts of uniquely mapping sequence reads. CNA plots were generated by plotting the ratio of unique sequence reads per bin, and applying a smoothing algorithm to generate integer-level copy-number values for each bin^61^,^62^,^63^. Cell clonality was assessed by comparing the location of break points of amplifications and deletions across the single-cell profiles.

### Culture and siRNA knockdown of VE-cadherin in NCI-H446 cells

NCI-1048 and NCI-446 SCLC cell lines (American Type Culture Collection) were authenticated (ampflstr, Applied Biosystems) and cultured in RPMI media (Life Technologies) containing 10% fetal bovine serum (Biowest) at 37 °C and 5% CO_2_. C8161 melanoma cells (a gift from Mary Hendrix) were cultured as above. Cell lines were authenticated using Promega Powerplex 21 System and routinely screened for mycoplasma within CRUK Manchester Institute's core facility.

Viral particles containing five shRNAs (Thermo Scientific, TRCN0000054088, TRCN0000054089, TRCN0000054090, TRCN0000054091 and TRCN0000054092) targeted against VE-cadherin cloned separately into the pLKO.1 lentiviral vector (Dharmacon, GE Lifesciences) and the pLKO.1 TRC Control vector (Addgene #10879) were generated by co-transfecting Lenti-X 293T (Clontech) cells with respective shRNA or control plasmids and pCMV-R8.91 and pMD2.G (a kind gift from Dr Orimo, Juntendo University, Tokyo, Japan) according to manufacturer's recommendations^48^. NCI-H446 cells were transfected with virus plus 6 μg per ml polybrene (Sigma) and selected with 1 μg per ml puromycin (Sigma) for ∼7 days. Polyclonal mixes for each shRNA plus control were generated and screened for knockdown of VE-cadherin by western blot.

### Network formation on matrigel

Six well tissue culture dishes were coated with 200 μl of Matrigel (BD Biosciences) and incubated at 37 °C for 30 min. NCI-H446, NCI-H446 shRNA VE-cadherin, NCI-H446 PLKO.1 non-targeting shRNA, H1048 and C8161 cells were seeded onto the plates at 2.5 × 10^5^ cells per well and VM networks were imaged by phase-contrast microscopy over 72 h within 4 randomly selected fields of view per well^7^.

### Cisplatin delivery and efficacy *in vivo*

NCI-H446 and H446 VE-cadherin KD cell xenografts were grown by s.c. injection of 1 × 10^6^ cells (0.1 ml of serum-free medium/matrigel 1:1) into the back mid-dorsal region of 6–8-week-old female NSG mice. Mice were housed in individually vented caging systems, maintained at a uniform temperature and humidity, set on a 12 h light/12 h dark environment. Individual mouse weights and tumour volumes (calculated as (length × (tumour width)^2^)/2) were measured three times a week, until a tumour volume of ∼250 mm^3^, when they were assigned to experiments. To assess cisplatin delivery animals were randomised into treatment groups (via a deterministic method without blinding) and received either a single dose of 7.5 mg per kg cisplatin i.p. or 10 ml per kg vehicle i.p., they were culled 1 h later via a Schedule I method. Tumours were half formalin-fixed and paraffin-embedded for analysis by IHC.

To assess the impact of VM on cisplatin/etoposide efficacy, animals bearing NCI-H446 or H446 VE-cadherin KD cell xenografts received either a single dose 5 mg per kg cisplatin i.p. on day 1 and 8 mg per kg etoposide i.p. on days 1, 2 and 3 or 5 ml per kg vehicle (0.9% saline+citric acid+NMP) on days 1, 2 and 3 i.p. Animals were culled via a Schedule I method when tumours reached 1,000 mm^3^. Tumours were formalin-fixed and paraffin-embedded for analysis by IHC. All procedures were carried out in accordance with Home Office Regulations (UK) and the UK Coordinating Committee on Cancer Research guidelines and by approved protocols (Home Office Project license no. 40-3306 and Cancer Research UK Manchester Institute Animal Welfare and Ethical Review Advisory Board) and in accordance with ARRIVE guidelines^64^.

### Data availability

All relevant sequencing data is uploaded to the European Genome-phenome Archive (EGA; https://www.ebi.ac.uk/ega/home) under the accession number EGAS00001001944.

## Additional information

**How to cite this article:** Williamson, S. C. *et al*. Vasculogenic mimicry in small cell lung cancer. *Nat. Commun.* **7,** 13322 doi: 10.1038/ncomms13322 (2016).

**Publisher's note:** Springer Nature remains neutral with regard to jurisdictional claims in published maps and institutional affiliations.

## Supplementary Material

Supplementary Information Supplementary Figures 1 - 8, Supplementary Tables 1 - 5

Peer Review
