# Supplementary material for: Vasculogenic mimicry in small cell lung cancer
Source: Nat Commun. 2016 Nov 9;7:13322. doi: 10.1038/ncomms13322 (PMC5105195; doi:10.1038/ncomms13322)
Supplement: Supplementary Information — Supplementary Figures 1 - 8, Supplementary Tables 1 - 5 [file ncomms13322-s1.pdf]

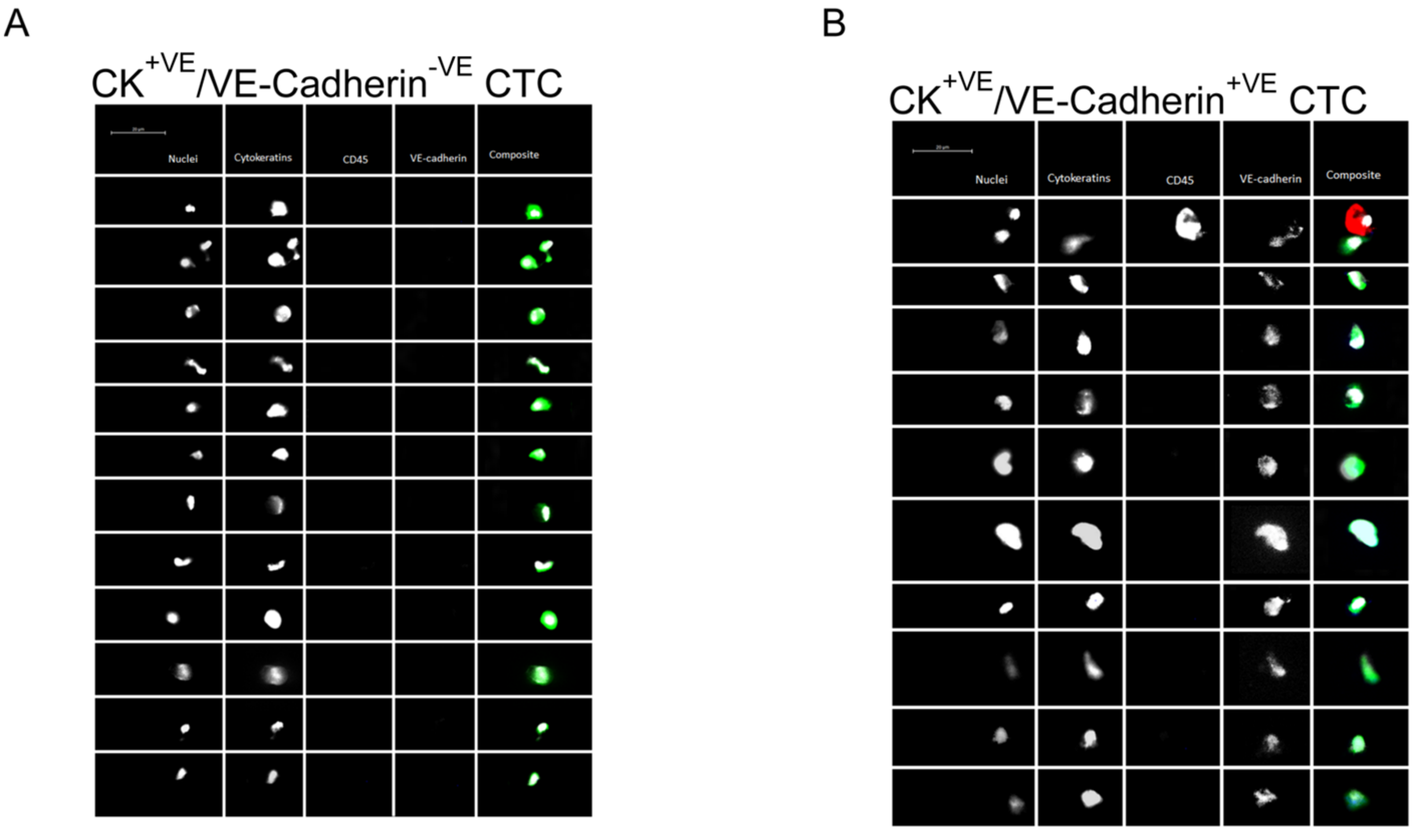

Supplementary Figure 1. CTC Gallery of representative images from ISET filters. A Representative images of DAPI^{+VE}/CK^{+VE}/VE-Cadherin^{-VE} CTC, **B** Representative images of DAPI^{+VE}/CK^{+VE}/VE-Cadherin^{+VE} CTC. Scale bar 20µm. Individual colour channels in greyscale, DAPI/Nuclei (first column), CK (second column) CD45 (third column) and VE-Cadherin (fourth column), pseudo coloured merged images (fifth column).

A

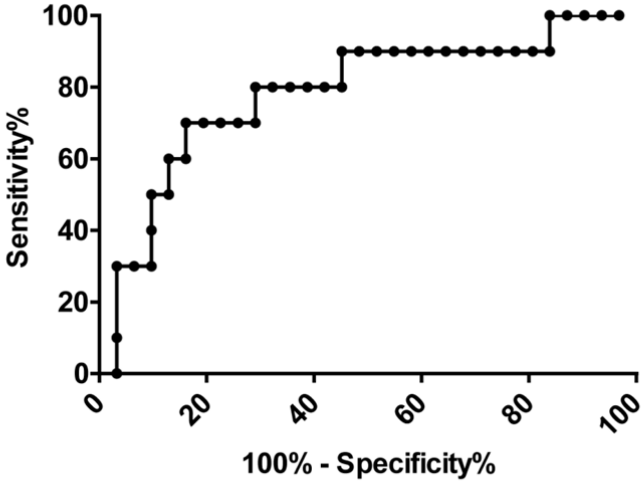

B

Area under the ROC curve	
Area	0.78
Standard Error	0.088
95% confidence inerval	0.61 to 0.96
P value	0.0076
Data	
Controls (Dead at 3years)	31
Patients (Alive at 3 years)	10

C

Cutoff	Sensitivity%	95% CI	Specificity%	95% CI
< 0.02584	0.0	0.0% to 30.85%	96.77	83.30% to 99.92%
< 0.03019	10.00	0.25% to 44.50%	96.77	83.30% to 99.92%
< 0.03414	30.00	6.67% to 65.25%	96.77	83.30% to 99.92%
< 0.03712	30.00	6.67% to 65.25%	93.55	78.58% to 99.21%
< 0.04035	30.00	6.67% to 65.25%	90.32	74.25% to 97.96%
< 0.05031	40.00	12.16% to 73.76%	90.32	74.25% to 97.96%
< 0.05740	50.00	18.71% to 81.29%	90.32	74.25% to 97.96%
< 0.05877	50.00	18.71% to 81.29%	87.10	70.17% to 96.37%
< 0.06019	60.00	26.24% to 87.84%	87.10	70.17% to 96.37%
< 0.06514	60.00	26.24% to 87.84%	83.87	66.27% to 94.55%
< 0.07542	70.00	34.75% to 93.33%	83.87	66.27% to 94.55%
< 0.08414	70.00	34.75% to 93.33%	80.65	62.53% to 92.55%
< 0.08822	70.00	34.75% to 93.33%	77.42	58.90% to 90.41%
< 0.09192	70.00	34.75% to 93.33%	74.19	55.39% to 88.14%
< 0.09734	70.00	34.75% to 93.33%	70.97	51.96% to 85.78%
**< 0.1056**	**80.00**	**44.39% to 97.48%**	**70.97**	**51.96% to 85.78%**
< 0.1144	80.00	44.39% to 97.48%	67.74	48.63% to 83.32%
< 0.1188	80.00	44.39% to 97.48%	64.52	45.37% to 80.77%
< 0.1207	80.00	44.39% to 97.48%	61.29	42.19% to 78.15%
< 0.1216	80.00	44.39% to 97.48%	58.06	39.08% to 75.45%
< 0.1384	80.00	44.39% to 97.48%	54.84	36.03% to 72.68%
< 0.1576	90.00	55.50% to 99.75%	54.84	36.03% to 72.68%
< 0.1635	90.00	55.50% to 99.75%	51.61	33.06% to 69.85%
< 0.1683	90.00	55.50% to 99.75%	48.39	30.15% to 66.94%
< 0.1718	90.00	55.50% to 99.75%	45.16	27.32% to 63.97%
< 0.1839	90.00	55.50% to 99.75%	41.94	24.55% to 60.92%
< 0.2038	90.00	55.50% to 99.75%	38.71	21.85% to 57.81%
< 0.2140	90.00	55.50% to 99.75%	35.48	19.23% to 54.63%
< 0.2161	90.00	55.50% to 99.75%	32.26	16.68% to 51.37%
< 0.2196	90.00	55.50% to 99.75%	29.03	14.22% to 48.04%
< 0.2253	90.00	55.50% to 99.75%	25.81	11.86% to 44.61%
< 0.2462	90.00	55.50% to 99.75%	22.58	9.54% to 41.10%
< 0.2757	90.00	55.50% to 99.75%	19.35	7.45% to 37.47%
< 0.2891	90.00	55.50% to 99.75%	16.13	5.45% to 33.73%
< 0.2901	100.0	69.15% to 100.0%	16.13	5.45% to 33.73%
< 0.3051	100.0	69.15% to 100.0%	12.90	3.63% to 29.83%
< 0.3238	100.0	69.15% to 100.0%	9.677	2.04% to 25.75%
< 0.3444	100.0	69.15% to 100.0%	6.452	0.79% to 2.42%
< 0.4306	100.0	69.15% to 100.0%	3.226	0.08% to 16.70%

Supplementary Figure 2. Receiver operating characteristic (ROC) curve analysis of SCLC tissue microarray VM scores A. ROC curve with sensitivity (%) against 100-specficity (%) for VM scores from 41 SCLC TMA patients and **B.** corresponding area under the ROC curve report **C.** Table of the sensitivity and specificity for a range of VM scores identifying a threshold with the greatest sensitivity and specificity of 11% (highlighted in bold font).

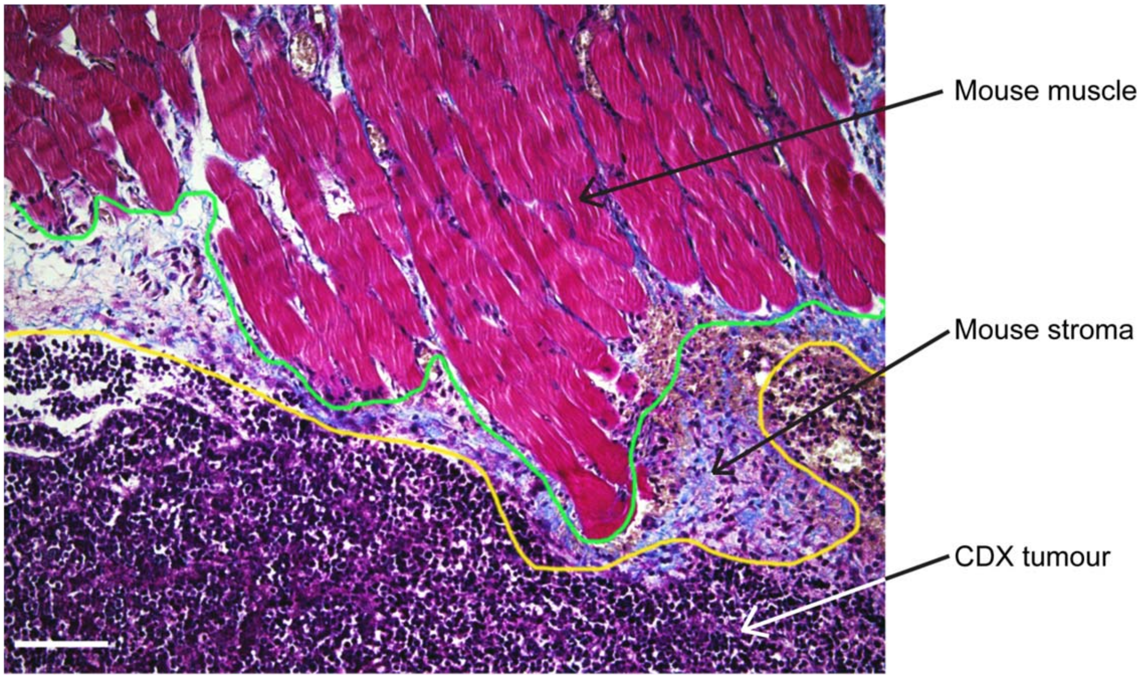

Supplementary Figure 3. Representative image of Masson trichrome (MT) staining of CDX tumour. MT was used to identify muscle (Pink) and stroma (Blue) and tumour (Dark Purple) regions used for controls in LCM analysis. Scale bar 100µm

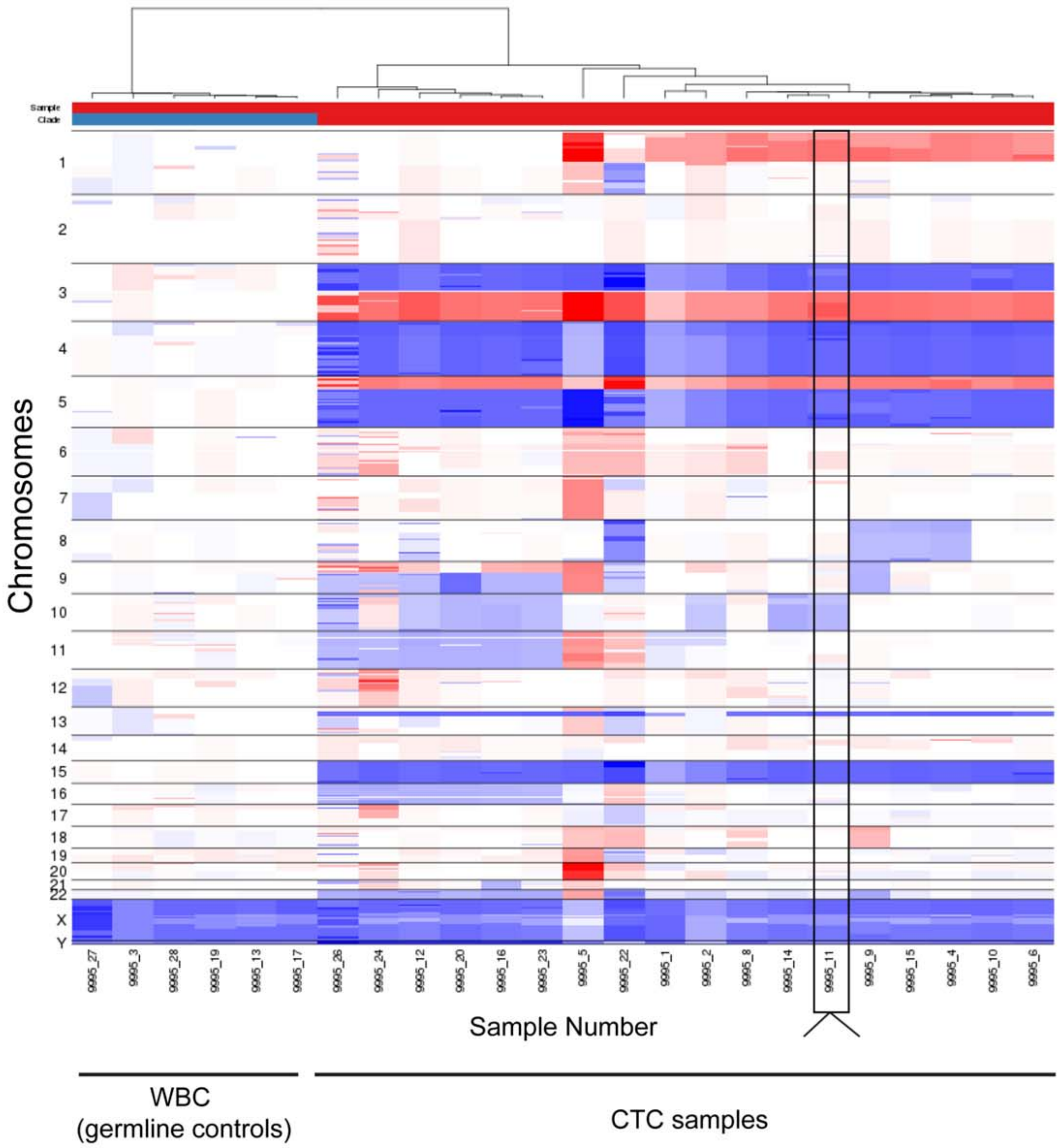

Supplementary Figure 4. Hierachical clustering of HD-SCA isolated CTCs. CNA profiles demonstrate all CTCs are clonally related to one another regardless of VE-Cadherin status, and are distinct to normal healthy germline control samples. A VE-Cadherin^{+VE} CTC highlighted with arrowhead.

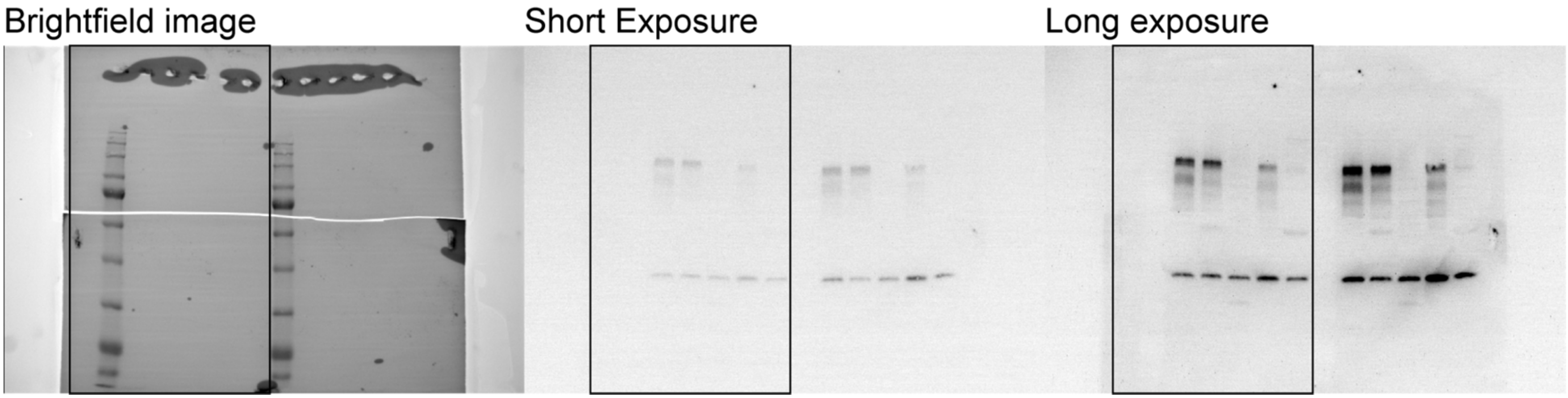

Supplementary Figure 5. Raw western blot scans used to generate Figure 7A. Black box indicate lanes shown in main figure

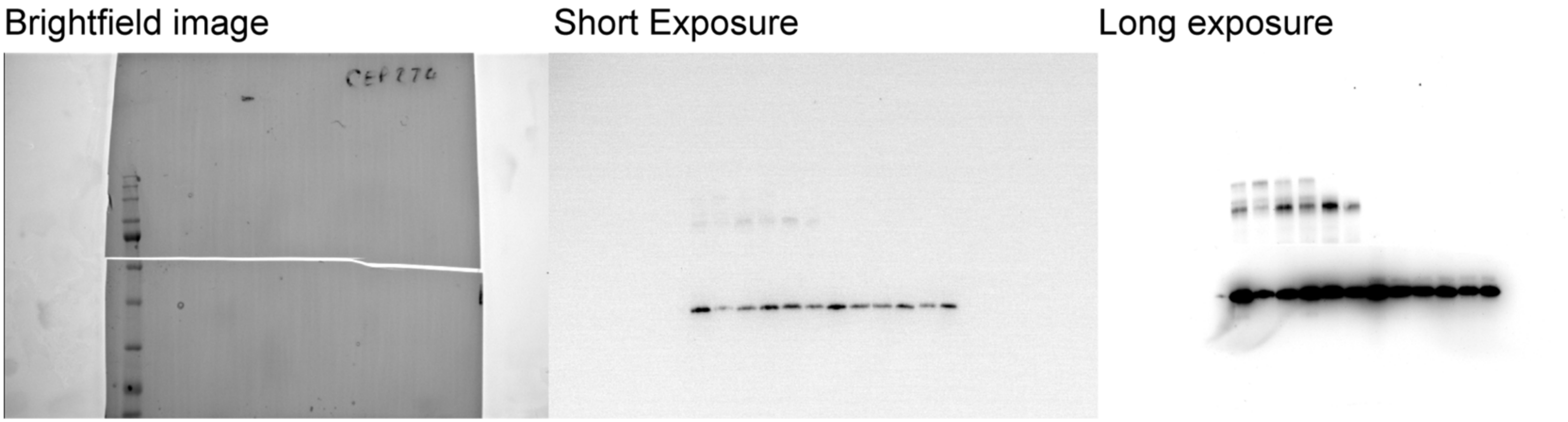

Supplementary Figure 6. Raw western blot scans used to generate Figure 7E

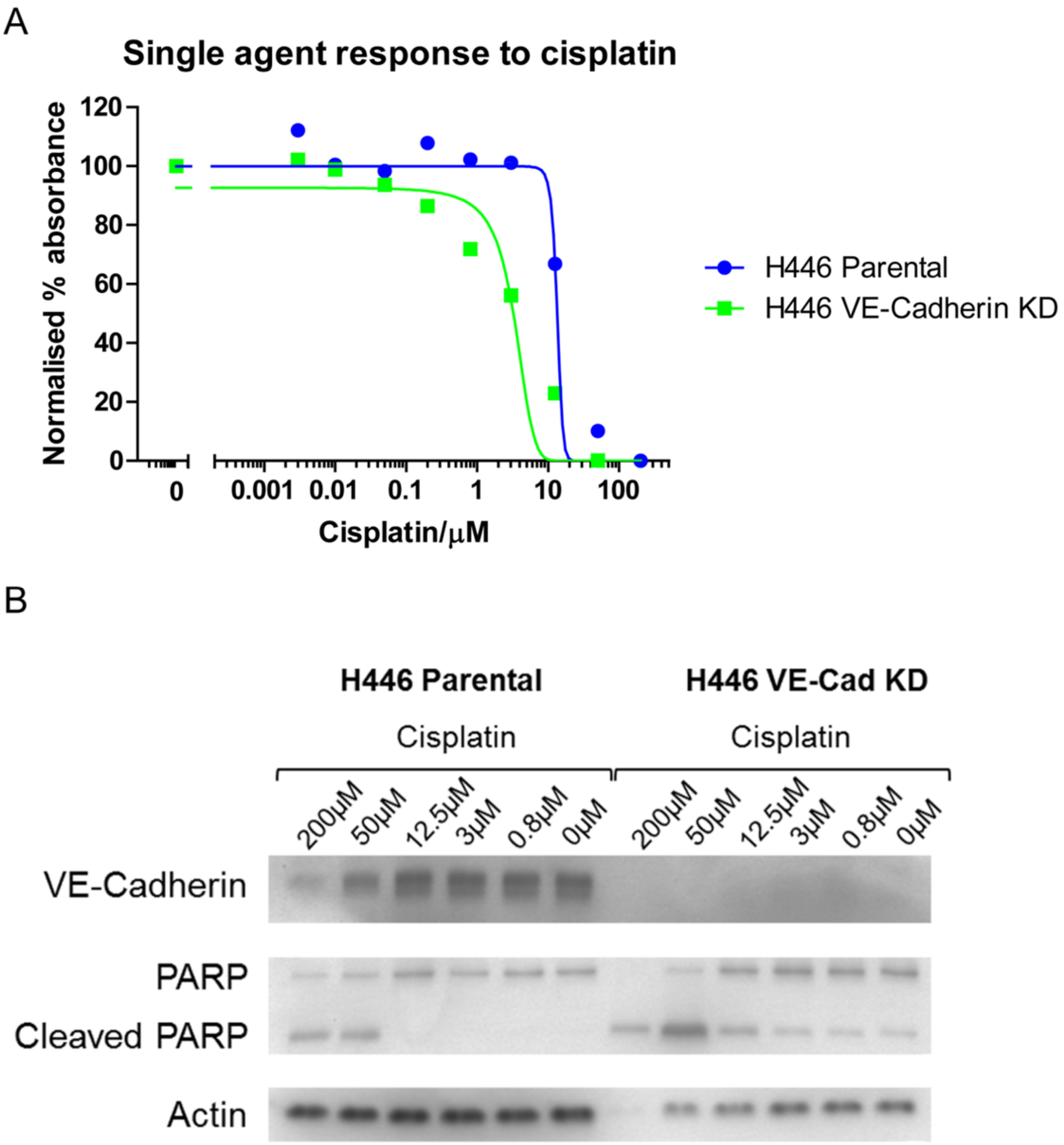

Supplementary Figure 7. Sensitivity of H446 and H446 VE-Cadherin KD to cisplatin. A. Cisplatin concentration response curve after 72h treatment in H446 parental and H446 VE-Cadherin KD cells (n=4). **B** Western blot of VE-Cadherin and cleaved PARP expression following 24h cisplatin treatment in H446 parental and H446 VE-Cadherin KD cells.

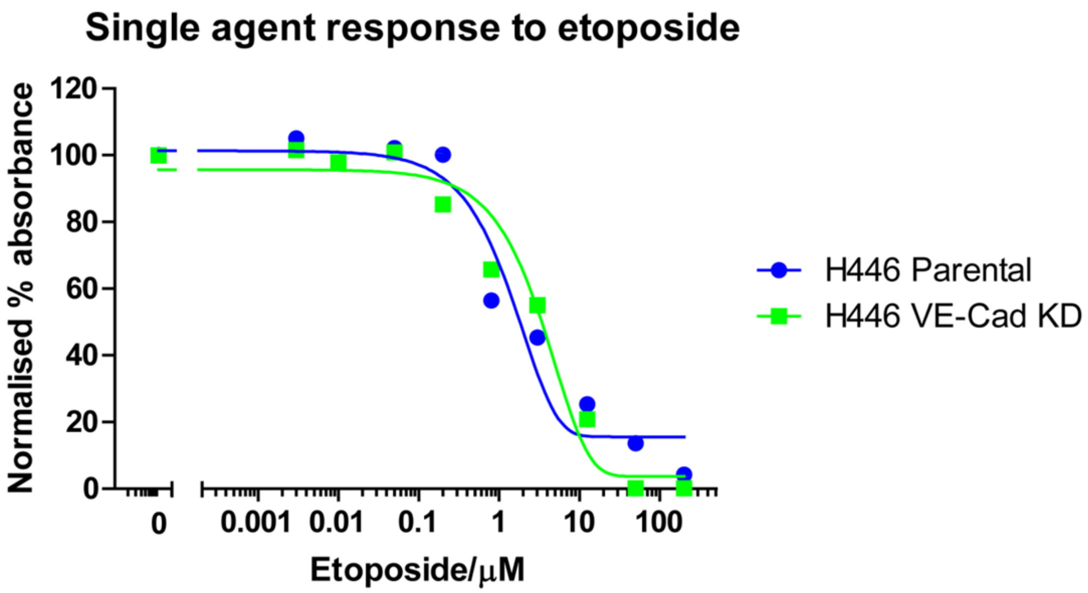

Supplementary Figure 8. Sensitivity of H446 and H446 VE-Cadherin KD to etoposide. Etoposide concentration response curve after 72h treatment in H446 parental and H446 VE-Cadherin KD (n=3).

	All patients (n=41)	Low TMA VM Score, ≤11% (n=17)	High TMA VM score, >11% (n=24)	
	Mean (Range)	Mean (Range)	Mean (Range)	p value
**VM score (%)**	15.5 (0 - 50)	5.8 (2.2 - 10.0)	22.3 (11.1 - 50)	0.025
**Overall Survival (Months)**	13.7 (0.0-27.9)	23.7 (0-70.4)	12.7 (6.3-19.2)	
	Mean (SD)	Mean (SD)	Mean (SD)	
**CD31+/PAS+ per core**	30.0 (17.7)	34 (20.2)	27 (15.1)	0.27
**Age (years)**	62 (37 - 82)	58 (37 - 72)	65 (51 - 82)	0.01
	Mean Units (SD)	Mean Units (SD)	Mean Units (SD)	
**Haematology**				
Haemoglobin (g/L)	128 (17)	127 (15.8)	128 (18.7)	0.95
Leukocytes (x10(9)/L)	10.8 (4.3)	10.8 (3.6)	10.8 (4.8)	0.94
Neutrophils (x10(9)/L)	8.2 (4.0)	8.3 (3.5)	8.1 (4.5)	0.9
Platelets (x10(9)/L)	355 (152)	360 (144)	349 (159)	0.94
**Biochemistry**				
Creatinine (umol/L)	82 (24)	78 (20)	85 (27)	0.5
Sodium (mmol/L)	134 (5.7)	134 (6.5)	134 (5.1)	0.81
Lactate dehydrogenase (IU/L)	772 (439)	682 (358)	833 (477)	0.42
Aspartate aminotrasferase (IU/L)	29 (11.7)	23 (4.1)	33 (13.2)	0.05
Alkaline phosphatase (IU/L)	84 (19.8)	78 (18.7)	88 (19.6)	0.19
Albumin (g/L)	39 (5.7)	41 (4.8)	37 (5.9)	0.06

Supplementary Table 1. Clinical Characteristics of the 41 LS SCLC patients whose lymph node biopsies comprise the tissue micro-array.

	Survival from diagnosis All stage			
	n	Median OS (months)	95% CI	p-value
**Age**				
<60	20	14.2	11.7 - 16.7	0.379
60 and over	21	13.2	0.0 - 27.9	
**Biochemistry***				
LDH <550	10	15.4	12.8 - 17.9	0.399
LDH >=550	15	11.7	0.0 - 26.1	
Na <132	12	7.8	0.0 - 19.1	0.034
Na >= 132	23	15.4	4.6 - 26.1	
Albumin <35	8	0.8	0.0 - 1.6	0.019
Albumin >=35	24	14.8	12.8 - 16.8	
**Haematology**				
Hb <11	7	13.2	0.0 - 46.0	0.956
Hb >=11	28	13.7	11.0 - 16.4	
WCC <=11	22	15.4	6.7 - 24.0	0.012
WCC >11	13	5.4	0.0 - 13.4	
Plt <=400	23	14.8	12.2 - 17.5	0.421
Plt >350	12	8	0.0 - 24.7	
**TMA**				
VM Low (<=11%)	17	23.8	0 - 70.4	0.025
VM high (>11%)	24	13.0	6.3 - 19.2	
Vessels per core <median	20	13.3	9.9 - 16.8	0.79
Vessels per core >median	21	14.2	7.4 - 21.0	

Supplementary Table 2. Univariate survival data for LS SCLC patients whose lymph node biopsies were subjected to the tissue microarray based analysis. Patients with a VM score >11% had significantly reduced 3 year survival compared to those with a VM score <11% (p=0.025). *Alk Phos, creatine and AST could not be assessed as too few samples were outside the range required to dichotomise.

Index	Name	Description	Total reads	No. reads aligned to human	% reads aligned to human	No. reads aligned to mouse	% reads aligned to mouse	No. reads aligned to both species	% reads aligned to both species	No. reads aligned to human only	% reads aligned to human only	% mouse DNA contribution (using Germline control as baseline)
1	CDX3$	VM Low	2776994	2440224	87.87	498257	17.94	442172	15.92	1998052	71.95	5.53
2	CDX3$	VM High	3349906	2724588	81.33	506853	15.13	437348	13.06	2287240	68.28	2.66
3	CDX3#	VM Low	1232864	1036602	84.08	187986	15.25	156494	12.69	880108	71.39	2.3
4	CDX3#	VM High	1713944	1384899	80.8	309129	18.04	213176	12.44	1171723	68.36	2.04
5	patient 3	germline	1920828	1535766	79.95	216040	11.25	199676	10.4	1336090	69.56	0
6	CDX3	CDX3 tumour	1928136	1218146	63.18	244344	12.67	191192	9.92	1026954	53.26	-0.48
7	Mouse	Mouse stroma	1186028	173052	14.59	1011245	85.26	150652	12.7	22400	1.89	
8	Mouse	Mouse muscle	1454088	196500	13.51	1236245	85.02	171160	11.77	25340	1.74	

Supplementary Table 3. Expanded analysis of the data presented in Figure 4C. Sequencing data from the 8 different samples used for Copy Number Analysis (CNA) shows that mouse DNA contribution ranges between 0.00 to 5.53%. All reads were aligned to both human and mouse genome separately.

Index	Name	Description	Total reads	No. reads aligned to human	% reads aligned to human	No. reads aligned to mouse	% reads aligned to mouse	No. reads aligned to both species	% reads aligned to both species	No. reads aligned to human only	% reads aligned to human only	% mouse DNA contribution (using Germline control as baseline)
1	CDX3(a)	Mouse MHC1^{-VE}/bulk tumour n=1	4917718	4644004	94.43	1448295	29.45	1355916	27.57	3288088	66.86	0.77
13	CDX3(a)	Mouse MHC1^{-VE}/bulk tumour n=2	4189188	3947985	94.24	1253751	29.93	1175768	28.07	2772217	66.18	1.25
2	CDX3(a)	Mouse MHC1^{-VE}/VE-Cadherin^{+VE} n=1	4033830	3798696	94.17	1148769	28.48	1070504	26.54	2728192	67.63	-0.2
14	CDX3(a)	Mouse MHC1^{-VE}/VE-Cadherin^{+VE} n=2	4069198	3787527	93.08	1232278	30.28	1136436	27.93	2651091	65.15	1.6
3	CDX3(a)	Mouse MHC1^{-VE}/VE-Cadherin^{-VE} n=1	544340	514440	94.51	153475	28.19	144248	26.5	370192	68.01	-0.49
15	CDX3(a)	Mouse MHC1^{-VE}/VE-Cadherin^{-VE} n=2	2241080	2107986	94.06	635247	28.35	596320	26.61	1511666	67.45	-0.33
4	CDX3(b)	Mouse MHC1^{-VE}/bulk tumour n=1	1248180	1171476	93.85	359416	28.8	337816	27.06	833660	66.79	0.12
16	CDX3(b)	Mouse MHC1^{-VE}/bulk tumour n=2	2113694	1947690	92.15	607947	28.76	571902	27.06	1375788	65.09	0.08
5	CDX3(b)	Mouse MHC1^{-VE}/VE-Cadherin^{+VE} n=1	406208	382329	94.12	115811	28.51	108158	26.63	274171	67.5	-0.17
18	CDX3(b)	Mouse MHC1^{-VE}/VE-Cadherin^{+VE} n=2	5004182	4695126	93.82	1559422	31.16	1462072	29.22	3233054	64.61	2.48
6	CDX3(b)	Mouse MHC1^{-VE}/VE-Cadherin^{-VE} n=1	2001928	1891304	94.47	552783	27.61	520302	25.99	1371002	68.48	-1.07
19	CDX3(b)	Mouse MHC1^{-VE}/VE-Cadherin^{-VE} n=2	2707154	2541460	93.88	811421	29.97	762772	28.18	1778688	65.7	1.29
12	patient 3	germline n=1	6898926	6499790	94.21	2016078	29.22	1890150	27.4	4609640	66.82	0.54
27	patient 3	germline n=2	4191444	3944256	94.1	1179335	28.14	1105202	26.37	2839054	67.73	-0.54

Supplementary Table 4. Sequencing data from the 10 different samples in duplicate used for Copy Number Analysis (CNA) following FACs sorting shows that mouse DNA contribution ranges between 0.00 to 2.48%. All reads were aligned to both human and mouse genome separately.

Key	Gene	Cytoband	SNV type	Annotation	Amino acid change	chr	position	ref	alt	normal_ref_count	normal_alt_count	normal_var_freq	tumour_ref_count	tumour_alt_count	tumour_var_freq	AAChange (based on refGene)	cosmic68	SNP138
chr17:7577535:C>A	TP53	17p13.1	nonsynonymous SNV	exonic	R117M,R90M,R210M,R249M	17	7577535	C	A	660	10	0.014925	359	225	0.385274	TP53:NM_001126115:exon3:c.G350T:p.R117M,TP53:NM_001126116:exon3:c.G350T:p.R117M,TP53:NM_001126117:exon3:c.G350T:p.R117M,TP53:NM_001276697:exon3:c.G269T:p.R90M,TP53:NM_001276698:exon3:c.G269T:p.R90M,TP53:NM_001276699:exon3:c.G269T:p.R90M,TP53:NM_001126118:exon6:c.G629T:p.R210M,TP53:NM_000546:exon7:c.G746T:p.R249M,TP53:NM_001126112:exon7:c.G746T:p.R249M,TP53:NM_001126113:exon7:c.G746T:p.R249M,TP53:NM_001126114:exon7:c.G746T:p.R249M,TP53:NM_001276695:exon7:c.G629T:p.R210M,TP53:NM_001276696:exon7:c.G629T:p.R210M,TP53:NM_001276760:exon7:c.G629T:p.R210M,TP53:NM_001276761:exon7:c.G629T:p.R210M	ID=COSM43871,COSM326724,COSM1649403,COSM326723;OCCURENCE=1(breast),1(haematopoietic_and_lymphoid_tissue),3(stomach),1(urinary_tract),1(pancreas),4(liver),1(oesophagus),14(lung),3(upper_aerodigestive_tract),2(large_intestine)	
chr17:7577565:T>C	TP53	17p13.1	nonsynonymous SNV	exonic	N107S,N80S,N200S,N239S	17	7577565	T	C	692	7	0.010014	403	240	0.37325	TP53:NM_001126115:exon3:c.A320G:p.N107S,TP53:NM_001126116:exon3:c.A320G:p.N107S,TP53:NM_001126117:exon3:c.A320G:p.N107S,TP53:NM_001276697:exon3:c.A239G:p.N80S,TP53:NM_001276698:exon3:c.A239G:p.N80S,TP53:NM_001276699:exon3:c.A239G:p.N80S,TP53:NM_001126118:exon6:c.A599G:p.N200S,TP53:NM_000546:exon7:c.A716G:p.N239S,TP53:NM_001126112:exon7:c.A716G:p.N239S,TP53:NM_001126113:exon7:c.A716G:p.N239S,TP53:NM_001126114:exon7:c.A716G:p.N239S,TP53:NM_001276695:exon7:c.A599G:p.N200S,TP53:NM_001276696:exon7:c.A599G:p.N200S,TP53:NM_001276760:exon7:c.A599G:p.N200S,TP53:NM_001276761:exon7:c.A599G:p.N200S	ID=COSM473434,COSM473433,COSM1649401,COSM473432,COSM44094;OCCURENCE=1(ovary),1(breast),1(stomach),4(haematopoietic_and_lymphoid_tissue),1(kidney),1(soft_tissue),4(urinary_tract),2(oesophagus),2(lung),2(large_intestine),1(central_nervous_system),1(endometrium)	
chr13:49039374:C>T	RB1	13q14.2	stopgain	exonic	R787X	13	4.9E+07	C	T	749	6	0.007947	236	396	0.626582	RB1:NM_000321:exon23:c.C2359T:p.R787X	ID=COSM23824,COSM1367318;OCCURENCE=1(ovary),1(prostate),1(large_intestine),2(central_nervous_system)	rs137853293
chr6:117662763:C>A	ROS1	6q22.1	nonsynonymous SNV	exonic	V1568L	6	1.2E+08	C	A	770	3	0.003881	492	253	0.339597	ROS1:NM_002944:exon29:c.G4702T:p.V1568L		
chr6:117714446:C>A	ROS1	6q22.1	nonsynonymous SNV	exonic	E401D	6	1.2E+08	C	A	744	11	0.01457	476	269	0.361074	ROS1:NM_002944:exon11:c.G1203T:p.E401D		
chr7:55260458:G>C	EGFR	7p11.2		splicing		7	5.5E+07	G	C	755	15	0.019481	483	287	0.372727			
chr17:59760687:T>C	BRIP1	17q23.2	synonymous SNV	exonic	K1240K	17	6E+07	T	C	754	7	0.009198	470	296	0.386423	BRIP1:NM_032043:exon20:c.A3720G:p.K1240K		
chr19:9065608:T>G	MUC16	19p13.2	synonymous SNV	exonic	R7280R	19	9065608	T	G	771	6	0.007722	592	151	0.20323	MUC16:NM_024690:exon3:c.A21838C:p.R7280R		

Supplementary Table 5 List of SNVs detected in the ctDNA of the patient's blood sample matched to that for HD-SCA analysis in Figure 7. Inclusion criteria required tumour variant allele frequency (VAF) to be greater than 0.025, for the fold difference between tumour VAF and normal VAF to be greater than 5 and for total read depth in tumour and normal samples to be greater than 200.
