# Supplementary material for: Vasculogenic mimicry in small cell lung cancer
Source: Nat Commun. 2016 Nov 9;7:13322. doi: 10.1038/ncomms13322 (PMC5105195; doi:10.1038/ncomms13322)
Supplement: Peer Review [file ncomms13322-s2.pdf]

PEER REVIEW FILE

Reviewers' comments:

Reviewer #1 (non-small cell lung cancer expert):

The manuscript by Metcalf et al. describes vasculogenic mimicry in SCLC. Overall the results are interesting, however a number of things need to be addressed.

• Second page of results (pages are missing in the manuscript): although CDX models are certainly interesting, they may not reflect the primary tumor, in that these are cells that have extravasated into circulation and expression of markers, such as VE-cadherin may be different for that reason. This should be discussed.
• Page 3 of results: authors state that VM high, VM low and CDX bulk are generically related. Not clear what is meant by this.
• Page 4 of results, end of first paragraph: the number of CTCs in the CellSearch is higher not lower.
• Page 5 of results: figure 6A shows bridges between cells. Authors call them VM networks. They look to me more like cellular extensions. How is VM defined ?
• Page 5 of results: another cell line (e.g. C8161) should be tested for VE-Cadherin knowckdown.
• Page 5 of results: authors state that there was a 7 day delay in tumor growth in tumor xenografts. This seems to be a very small effect, albeit significant. Also, if VM is related to metastasis and VE-Cadherin is correlated with VM score, how would this influence tumor growth in a xenograft model ? a model of metastasis formation would be more appropriate.
• Page 5 of results: it is not clear from these results if reduction in cisplatin-DNA adducts after VE-Cadherin knockdown is really due to drug penetration or some other mechanism related to DNA damage induction.
• Discussion: patients with limited disease are usually defined as those who are candidates for chemo-radiation, not resection. Apparently there were a few patients who were resected in the 41 case series. Suppl. Table 1 does not capture this information, nor does it capture important prognostic information such as performance status, gender, and treatment.
• Discussion: the reported expected 5-year survival in resectable SCLC is higher than 12%. Ref. 26 is a 1999 meta-analysis of PCI, and ref. 27 is an old paper from 2002. The inference that low VM score might be related to increased survival, based on these assumptions is incorrect.
• Fig. 1B: what are the capital letters on top of the graph ?
• Fig.2C: please add the number of patients at the main time points, and the censored patients in the graph.

Reviewer #2 (CTCs expert):

The paper by Metcalf and coworkers reports that many primary small cell lung cancers (SCLC) have tumor-derived vascular lining cells noted by their expression of cytokeratin VM, an adverse prognostic marker. VM positive vessels are also seen in virtually all mouse xenografts that are derived from human SCLC circulating tumor cells (CTCs). In such xenografts, presence of VM positive vessels paradoxically improves vascularity, drug access and tumor response, despite its characterization as an adverse prognostic marker. In studying the primary CTCs from patients with SCLC, the authors note that all patients have subsets of CTCs that expresse VE-Cadherin, a marker of endothelial cells, which the authors attribute to the transdifferentiation of the primary SCLC cells. However, the genomic (copy number alterations, CNA) within these CTCs and vimentin-rich areas of the tumor are different from those of the bona fide tumor cells. The authors conclude that the transdifferentiation of tumor cells into a vasculogenic cell fate has both positive and negative consequences for the progression and drug responsiveness of SCLC.

All together, this is an interesting topic, and the ability of tumor cells to create false vascular spaces is of considerable interest. While the paper addresses multiple aspects of this question, each one is presented in a rather superficial way, with significant technical concerns. The authors are encouraged to provide a deeper characterization of each observation and adjust the strength of their conclusions.

Specific concerns include the following:

1. CTCs were isolated by two different methods: size based filtering and high throughput imaging of unprocessed blood cells. It is not clearly stated which technology was used where. A potential concern about size based filtering of small cell lung cancer is that these are presumably small cells. Are they sufficiently larger than blood cells to allow this purification. What are the sizes of the CTCs and what is the purification efficiency?

2. The fact that there are different gene copy alterations (CNA) for bona fide tumor cells and for VE-positive CTCs is of significant concern. The authors conclude that the vascular transdifferentiating tumor cells originate a different primary population of tumor cells. That is a major conclusion indeed, but it is not supported by sufficiently credible data. A deeper analysis (not just CNA but specific mutation calls) would be critical here. Are there sufficient controls (with similarly processed normal cells) to be sure that the single cell CNA abnormalities are genuine and not artifactual?

3. How are we sure that VE-positive CTCs are indeed tumor-derived (beyond the conflicting CNA data). The cells have low cytokeratin (CK). On what basis are they thought to be tumor

derived CTCs as opposed to some nonmalignant endothelial cell type?

4. The quality of the CTC imaging is poor. How is VE cadherin expression quantitated? All cells shown in Figure 1A seem to have positive CD45 staining (a marker of hematopoietic cells). In fact all stains are positive for all immunofluorescence stains: the authors should present quantitative data on immunofluorescence scoring, controls and criteria. The Y axis in figure 1a is not labeled: are these different cells from different patients? In addition, instead of showing a few selected cells, they should show a low resolution image of a filter or epic slide, so that background signal in neighboring cells can be assessed.

5. The quality of the staining of tumor vascular structures is poor (Figure 2a). Better resolution is required. There seems to be purple material throughout the tumor slide, rather than a discrete vessel-like structure (is it background staining?). Both positive and negative staining controls should be shown.

6. A key point is that the vascular lining cells of human xenografts are human, rather than mouse-derived. There are human-specific antibodies available to distinguish human from mouse cells, that could make that case much more strongly and convincingly.

7. The authors imply but do not explain a VM score in tumors. How is that derived? If VM structures are present in 9/10 xenograft models, how is the score applied. How quantitative is the score, and is the single negative tumor totally negative? Are there other tumor type xenografts in which VM structures are absent (ie negative control)?

8. Just like VE-CTCs with different CNA compared with tumor CTCs, it is concerning that VM-low regions of tumor xenografts have different genetic CNA than VM-high regions. Is this true for all cases and how does the tumor section CNA relate to the CNA from matched CTCs? Are these consistent within individual patients? Across different patients, it would be strange if in every case, vasculogenic tumor cells originate from a separate tumor precursor that can be easily separated from the bulk tumor cells simply by their different CNA. Again, this calls into question the accuracy of the single cell CNA data and the possibility of technical artifacts.

9. The authors conclude that vascular mimicry can be good or bad in that it is associated with a worse clinical prognosis, but appears to reduce tumor hypoxia and improve drug access. These are interesting concepts, but they need to be based on more solid primary data.

Reviewer #3 (vascular mimicry expert):

Metcalf et al in the manuscript entitled "Vaculogenic Mimicry in Samll Cell Lung Cancer" provided solid evidence for the first time that a subpoluation of VE-cadherin-expressing CTCs exists, which drives VM in vivo in the CDX model, and that a VE-cadherin-expression tumor subset exhibits VM capacity in patient samples, which correlates inversely with disease survival. The findings are exciting and significant, because they provide biological rationale for developing therapeutic strategies targeting VM in the treatment of SCLC. The experimental design is logical and scientifically sound. The aproaches and statistical analyses used are appropriate. The analyses of patient-derived materials/ cells as well as the use of biologically relevant animal models significantly strengthen the study. Only minor comments are provided below:

1. Abbreviations are not spelled out at first mentioning, which makes it difficult to follow the manuscript.
2. How are limited vs. extnesive stage diseases defined?
3. The clonality of the VE-cadherin-non-expressin,g but not the VE-Cadherin-expressing, CTC population as revealed by the CNA anaylses is intriguing. It would be helpful to discuss further the significance of this obsevration and how it relates to disease progression.
4. Fig.1A: Additional labeling to highlight indiviual cell type illustrated in the panels; e.g. WBC, VE-Cadherin+ CTC, VE-cadherin- CTC, would be helpful
5. Fig. 1B: What do the 'E's" and 'L's" on top of the graph mean?
6. Fig. 2B: It is not clear if nuclear counter stain was used as the tumor cells surrounding the PAS+ channels are dissifult to appreciate

Reply to Reviewers' comments:

We thank the Reviewers and Editor for their comments, which seem to be mostly positive about our manuscript. Before the point by point rebuttal to each reviewer, we summarise the additional data added to the revised manuscript and general alterations resulting from the initial review process.

We have,

- Included page numbers and line numbers to aide replying to reviewer's comments.
- Clarified that as SCLC routinely presents with extensive disease CDX reflect a true representation of the clinical setting.
- Clarified that species specific antibodies were used throughout to ensure staining and isolation of human cells only.
- Clarified that bioinformatics pipelines accurately detect mouse reads and remove these from analysis ensuring only human DNA profiles are studied.
- Removed arguments relating to sub-clonal populations resulting in VM formation in preliminary data, focussing instead on our main intention, the demonstration of a human tumour origin of VM vessels and a tumour origin of VE-Cadherin positive CTCs.
- All figures have been regenerated at much higher resolution as requested.

Amended and additional data:

Figure 1 and Supplementary 1 include a CTC gallery of VE-Cadherin positive and negative cells as enriched by ISET filtration. We have also included absolute CTC counts demonstrating that VE-Cadherin positive CTCs are a rare subpopulation of detected CTCs.

Figure 2, as requested Kaplan Meier survival analysis of our TMA data now includes absolute patient numbers at major time points, and we have increased the size of censored data point markers for clarity.

Figure 3C is amended to include sequential section staining of Masson Trichrome to demonstrate that there was no mouse stroma invasion around areas of VM, supporting the human origin of VM vessels.

Figure 4 has been amended to clarify our arguments. We now only focus on the first time demonstration using genomic methods that VE-Cadherin positive VM vessels are of a human origin as evidenced by lack of mouse contaminating DNA reads.

New Figure 5 and Supplementary Table 4. To augment the original laser microdissection data, we FACs sorted CDX3 populations, depleting for mouse cells and sorting VE-Cadherin positive and negative cell subpopulations. We demonstrate both VE-Cadherin positive and negative cells have similar CNA gains and losses, and further demonstrate the presence of a

previously reported TP53 mutation in all tumour samples in this study of VM, further demonstrating VE-Cadherin positive CDX cells have a SCLC human origin.

New Figure 6, Supplementary Figure 3 and Supplementary Table 5. We now provide new CTC analysis using the HD-SCA platform, demonstrating VE-Cadherin positive and VE-Cadherin negative CTCs have a common clonal origin. CNA alterations demonstrate RB1 loss, consistent with SCLC. Analysis of circulating tumour DNA (ctDNA) from parallel blood samples from the same patient show matching CNA alterations to VE-Cadherin positive and VE-Cadherin negative single CTCs and targeted sequencing of this matched ctDNA revealed clonal *TP53* and *RB1* mutations, supporting the SCLC origin of VE-Cadherin positive/CK positive CTCs.

New Figure 7. Improved VE-Cadherin KD in H446 confirmed and magnified differences in the data submitted in the original manuscript. Sustained VE-Cadherin KD *in vivo* resulted in a greater reduction in VM vessel formation. Furthermore, improved VE-Cadherin KD led to significant greater latency before exponential tumour growth.

New Figure 8 and Supplementary Figure 4. We have included experiments assessing cisplatin sensitivity *in vitro* H446 cells with and without VE-Cadherin KD. VE-Cadherin KD cells were more sensitive to cisplatin compared to control. These *in vitro* data argue for an interpretation of the *in vivo* studies whereby VM reduces intra-tumoural cisplatin delivery rather than reduced intracellular processing of cisplatin.

New Figure 9 and Supplementary Figure 5. We have included a new *in vivo* efficacy study demonstrating that despite reduced delivery of cisplatin in H446 VE-Cadherin xenografts, tumours are more sensitive to cisplatin-etoposide, the standard of care for SCLC. We also demonstrate that *in vitro*, H446 and H446 VE-Cadherin KD cells are equally sensitive to etoposide.

All reviewers' specific comments are addressed in the following point-by-point reply.

Reviewer #1 (lung cancer expert):

The manuscript by Metcalf et al. describes vasculogenic mimicry in SCLC. Overall the results are interesting, however a number of things need to be addressed.

Second page of results (pages are missing in the manuscript): although CDX models are certainly interesting, they may not reflect the primary tumor, in that these are cells that have extravasated into circulation and expression of markers, such as VE-cadherin may be different for that reason. This should be discussed.

This is an interesting concept the Reviewer raises. Access to primary SCLC patient material is challenging, however we have previously demonstrated that CDX models mirror patient tumour specimen histology and cytology with respects to classic morphological features, expression of classic SCLC markers and to patient's response to chemotherapy ***(1)****. Unfortunately due to the limited material we receive from matched patient biopsies, we are unable to determine matched VE-Cadherin expression in tumour specimens and the TMA we used for VM vessel score was from limited stage patients and from loco-regional lymph nodes rather than primary tumour. However, all diagnostic markers we have assessed to date (CD56, Cytokeratin, Synaptophysin, Chromogranin) have shown equivalent staining*

patterning and intensity in CDX and matched patient biopsy material and include further details of the CDX/primary patient material comparison.

We have amended the text (Page 5 lines 18-23 and Page 6 lines 20-24) to reflect these points. Of note however, if CTC derived CDX did represent an aggressive subpopulation of the primary tumour, SCLC is so very highly metastatic it does not seem likely that only a minor subclone in the primary tumour is wholly responsible for disease progression and as such, that CDX are totally unrepresentative of primary tumours. Moreover, CDX are of value in this study as they may better represent the metastatic tumours that are lethal to the patient.

CDX facilitate the study of VM as endothelial vessels are murine and therefore easily distinguished from human VE-Cadherin positive VM vessels. Recent elegant studies of VM in breast cancer published in Nature Medicine used cell lines, which we argue are less representative of the patient (2).

Page 3 of results: authors state that VM high, VM low and CDX bulk are generically related. Not clear what is meant by this.

We thank the reviewer for their point, but we did not use the term generically. We presume the reviewer meant genetically?

Previous studies have demonstrated VM vessels are tumour derived in origin, either by the expression of unique features such as melanin containing melanosomes for melanoma (3), or through the expression of transgenes in implanted tumour cells (2). However, in this study, we did not have an appropriate pre-existing specific tag or phenotypic characteristic to monitor. As such we sought to demonstrate that the VM vessels were human tumour derived through demonstration of human genomes. Mutation profiles in VM high and VM low regions of CDX are inconsistent with non-cancerous human tissues, i.e., .demonstrating CNA profiles with considerable gain and losses typical of SCLC after removal of contaminating murine DNA reads bioinformatically (see amended Figure 4C and Supplementary Table 3). We added weight to this argument with new data, whereby CDX3 was dissociated to single cells, labelled with human VE-Cadherin and anti-mouse MHC1, sorted for VE-Cadherin positive and negative human cells, CNA profiles assessed, and targeted sequencing performed.

CNA profiles, consistent with those previously reported, along with the presence of a previously identified TP53 mutation in this CDX model were identified (1), confirming cells were tumour and genetically related to CDX3 bulk tumour.

This ‘cleaner’ sorting experiment confirmed the tumour origin of VE-Cadherin expressing human cells. This new data is described on page 8 line 11- page 9 line 2 and presented in New Figure 5 and Supplementary Table 4. We described the evidence for the tumour origin of VM networks in SCLC in the original Discussion section, however, we agree that an earlier explanation would be useful in the appropriate Results section which we have now added (Page 7 line 12- 17).

Page 4 of results, end of first paragraph: the number of CTCs in the CellSearch is higher not lower.

We thank the reviewer for this observation, two different scales were used to report CTC counts from CellSearch and CTC counts from HD-SCA methodology (per 7.5ml and per ml respectively). We can see why this would confuse the readers and we have amended the text to report CTC counts per ml of blood for easy comparison across studies.

Page 5 of results: figure 6A shows bridges between cells. Authors call them VM networks. They look to me more like cellular extensions. How is VM defined?

We thank the reviewer for their comment. The in vitro vessel formation assay on matrigel is the 'classic' gold standard experiment for assessing the ability of endothelial cells, and more recently VM competent cells, to form de novo networks. These reductionist experiments are typically used to demonstrate VM network formation potential in a closed system without the presence of confounding cell types, such as endothelial cells.

We therefore adopted this in vitro approach as the 'gold standard' to initiate the subsequent and more physiologically relevant in vivo studies. Nevertheless, previous studies in VM demonstrated that these in vitro networks are not just cellular extensions resulting from low density plating of any cell culture, but in fact are hollow, perfusable networks formed by cells capable of VM in vivo (4). We have included an extended description of these experiments in the text, along with appropriate references (page 10 lines 10-14).

Page 5 of results: another cell line (e.g. C8161) should be tested for VE-Cadherin knockdown.

We thank the Reviewer for this point, something we are actively exploring. The other 8 SCLC cell lines we assessed lacked VE-Cadherin expression and did not form VM networks in vitro. Our CDX models do express VE-Cadherin and do have VM potential, however to date modulation of gene expression with shRNA in these primary cells has proven challenging but is the subject of our future research programme.

The impact of VE-Cadherin knockdown on VM in C8161 melanoma cells was reported by Hendrix et al 2001 (4). We have now included a description of these experiments, along with the reference in the text (page 10 lines 14-15).

Page 5 of results: authors state that there was a 7 day delay in tumor growth in tumor xenografts. This seems to be a very small effect, albeit significant. Also, if VM is related to metastasis and VE-Cadherin is correlated with VM score, how would this influence tumor growth in a xenograft model? A model of metastasis formation would be more appropriate.

We thank the reviewer for their comments. Since submission, we have improved the VE-Cadherin KD in H446 using the same shRNA but with an improved selection for shRNA expressing cells. We now see a more profound increase in latency of tumour growth (23 days difference in median time to 200mm^2) and a more significant difference between the groups showing that VM has a physiological impact. The updated data is reported on page 10 line 19 onwards in the results section and in Figure 7. We add discussion of this latency effect on page 14 lines 18-23, suggesting that without VM, a longer time is required to recruit mouse vessels into the xenograft that deliver the oxygen and nutrients required to sustain exponential tumour growth. This is consistent with the equivalent growth rates in KD and

parental control H446 tumours once tumours initiate growth (Figure 7D(i)), rather than a cell autonomous difference in tumour cell doubling time.

The impact of VE-Cadherin and VM on SCLC metastases is the subject of a future study. Our CDX models do not routinely metastasise prior to primary tumours coming to size and sacrifice of the mouse. We are attempting to develop new protocols to address this limitation.

Page 5 of results: it is not clear from these results if reduction in cisplatin-DNA adducts after VE-Cadherin knockdown is really due to drug penetration or some other mechanism related to DNA damage induction.

The reviewer makes a good point. In response, we have included new data showing H446 VE-Cadherin KD cells have a reduced IC_{50} *values for cisplatin in vitro so are in fact more sensitive to cisplatin (Supplementary Figure 4). This sensitivity is seen in vivo, with H446 VE-Cadherin KD tumours showing increased tumour response and improved survival following treatment (Figure 9A and B).*

These in vitro and in vivo data are consistent with an interpretation that the differences in cisplatin-DNA adducts seen for H446 parental and H446 VE-Cadherin KD xenografts result from physiological parameters (such as vasculature) rather differential cellular 'handling' of cisplatin. This new data is reported and discussed on page 11 line 19 - page 12 line 2, page 12 lines 5 - 23 and page 14 line 24-page 15 line 6).

Discussion: patients with limited disease are usually defined as those who are candidates for chemo-radiation, not resection. Apparently there were a few patients who were resected in the 41 case series. Suppl. Table 1 does not capture this information, nor does it capture important prognostic information such as performance status, gender, and treatment.

We thank the reviewer for highlighting the inadequacy of the terminology we previously used in our description of patients as having 'resectable limited stage disease'. The patients in the 41 case series whose biopsies were analysed for VM vessels had limited stage disease defined as having disease which can be encompassed within a radiotherapy field, i.e. patients who are candidates for chemo-radiation as the reviewer describes. The word resectable has been removed from the text as none of the patients underwent surgical resection, this information was not included in Supplementary Table 1. Although data were available on haematology and biochemistry values, the clinical annotation on these 41 patients did not include additional data on treatment received and performance status at the time of receipt of treatment and so no amendment to Table 1 was possible.

Discussion: the reported expected 5-year survival in resectable SCLC is higher than 12%. Ref. 26 is a 1999 meta-analysis of PCI, and ref. 27 is an old paper from 2002. The inference that low VM score might be related to increased survival, based on these assumptions is incorrect.

We thank the reviewer for this insight which raises three issues to be addressed.

Firstly, as described above, the patients included in this study had limited stage disease rather than resectable disease. As such, the references included, document the survival of

patients with limited stage SCLC (rather than SCLC treated with surgical resection) in which setting the 5 year survival is expected to be less than 15%. The text is therefore adjusted to reflect this.

Secondly, the point is taken that the references are from 5 year survival published in studies in 1999 and 2002. These were selected as they reported 5 year survival in large number of limited stage SCLC patients. However, a more contemporaneous reference has been included to support the five year survival rates in the setting of limited stage SCLC. In this report, from the US National Cancer Institute Survival Epidemiology and End Results analysis, patients with SCLC defined as having 'regional disease', the five year relative survival was 14.7% (5). The text is amended to reflect this updating on page 13 lines 16-18.

*Thirdly, the reviewer questions 'the inference that low VM score might be related to increased survival, based on these assumptions. The data reported in this study demonstrate that patients with a low VM score have an improved survival compared with patients with a high VM score. However, this only acts to generate the hypothesis of causality. We acknowledge that the wording in the manuscript may be misleading and makes inferences of causality, where no inference is intended. Main text has been amended accordingly adjusted to "*A prospective study will be essential to confirm the negative impact of VM on SCLC prognosis and tractable surrogate models are needed to interrogate the molecular regulation of VM and investigate further its impact on SCLC biology and response to treatment*." (Page 13 line 18-20)*

Fig. 1B: what are the capital letters on top of the graph?

These letters signify whether the corresponding blood sample was derived from a limited stage patient (L) or an extensive stage patient (E). This information was in the figure legend but for increased clarity we have now added this information as a key in Figure1B itself.

Fig.2C: please add the number of patients at the main time points, and the censored patients in the graph

We thank the reviewer for highlighting this and have improved the figure accordingly. This data has now been added as a table below the Kaplan Meier plot (Figure 2C). Censored patients were highlighted as vertical dashes on the survival curves according to convention. These have been increased in size for clarity

Reviewer #2 (CTC expert)

The paper by Metcalf and co-workers reports that many primary small cell lung cancers (SCLC) have tumor-derived vascular lining cells noted by their expression of cytokeratin VM, an adverse prognostic marker. VM positive vessels are also seen in virtually all mouse xenografts that are derived from human SCLC circulating tumor cells (CTCs). In such xenografts, presence of VM positive paradoxically improves vascularity, drug access and tumor response, despite its characterization as an adverse prognostic marker. In studying the primary CTCs from patients with SCLC, the authors note that all patients have subsets of CTCs that express VE-Cadherin, a marker of endothelial cells, which the authors attribute to the transdifferentiation of the primary SCLC cells. However, the genomic (copy number alterations, CNA) within these CTCs and vimentin-rich areas of the tumor are different from those of the bona fide tumor cells. The authors conclude that the transdifferentiation of tumor

cells into a vasculogenic cell fate has both positive and negative consequences for the progression and drug responsiveness of SCLC.

All together, this is an interesting topic, and the ability of tumor cells to create false vascular spaces is of considerable interest.

While the paper addresses multiple aspects of this question, each one is presented in a rather superficial way, with significant technical concerns.

The authors are encouraged to provide a deeper characterization of each observation and adjust the strength of their conclusions.

We were disappointed that this reviewer found our research superficial. In response we have included substantially more data and improved all pre-existing figures to strengthen and clarify our arguments, providing a more in-depth analysis of VM in SCLC.

Specific concerns.

1. CTCs were isolated by two different methods: size based filtering and high throughput imaging of unprocessed blood cells. It is not clearly stated which technology was used where. A potential small cells. Are they sufficiently larger than blood cells to allow this purification. What are the sizes of the CTCs and what is the purification efficiency?

We thank the reviewer for their comment. We routinely use a variety of CTC technology platforms to address specific and diverse questions relating to CTC biology, as no single platform delivers answers for all tumour types. All platforms have advantages and limitations.

Although we felt this was stated in the submission, we have now clarified further which platform was used for each experiment conducted (revisions made on pages 5 lines 1 -23, Page 9 line 6- page 10 line 6).

Pertinent to this point, whilst ISET filtration allows identification of the extremely rare VE-Cad/CK co-expressing cells (Figure 1B), in our hands this technology is limited in its ability retrieve cells for downstream molecular analysis. To overcome this technical issue, molecular analyses of VE-Cad positive and negative CK positive CTCs were carried out using the HD-SCA platform. We respectfully suggest that the multiple approaches used, all consistent with VM in cells of tumour origin are not superficial but rather confirm our hypothesis that SCLC cells can undergo VM. This conclusion is more comprehensively supported in the revised manuscript.

With respects to size based isolation of SCLC, SCLC tumour cells are indeed small but this is relative to other tumour cell types. Estimates of mean SCLC cell size are 12.5µM – 14-1µM (6) which is greater than the 8 µM pore size used on ISET filters. As such, whilst yield may not be 100%, size based enrichment of SCLC has been used by others and ourselves previously to assess SCLC CTC phenotypes (7). This information on SCLC cell size is now added in the results section, page 5 line 5-8.

2. The fact that there are different gene copy alterations (CNA) for bonafide tumor cells and for VE-positive CTCs is of significant concern.

The CNA data were generated from samples that were bar coded and logged using our laboratory information management system (LIMS) and sample preparation and bioinformatic analysis carried out using established and published protocols (1, 8, 9).

It is unlikely that the CNA patterns are artefactual since this type of analysis encompasses multiple reads across the entire genome and our previous analysis has shown essentially 'flat' genomes for a variety of controls including non-cancer cells such as single leukocytes (1, 9). For LCM analysis, we have focussed instead on the fact that VE-Cadherin positive VM vessels have human genomes as evidenced by the species origin of the reads (Mouse DNA contribution ranged between 0 and 5.53%, Supplementary Table 3). This data has now been moved from the supplementary data to the main figure as Figure 4C.

To support the conclusions from the LCM data, we carried out additional experiments, sorting CDX3 by FACS to deplete mouse cells, and sort cells based on VE-Cadherin expression. Again human genomes were detected (murine DNA contribution ranged between 0 and 2.48%, Supplementary Table 4), characteristic SCLC CNA profiles seen (Figure 5C) and targeted sequencing revealed our previously reported TP53 mutation in this CDX model (1).

Furthermore in the current analysis neither the matched germline sample from patient 3 nor the white blood cell showed any obvious deviations in genome wide copy number nor harboured a TP53 mutation (Figure 5C, 5D 6B and 6C), further demonstrating the specificity of our analyses.

3. The authors conclude that the vascular transdifferentiating tumor cells originate a different primary population of tumor cells. That is a major conclusion indeed, but it is not supported by sufficiently credible data. A deeper analysis (not just CNA but specific mutation calls) would be critical here. Are there sufficient controls (with similarly processed normal cells) to be sure that the single cell CNA abnormalities are genuine and not artefactual?

On what basis are they thought to be tumor derived CTCs as opposed to some non-malignant endothelial cell type?

In keeping with the reviewer's request, we have also included specific mutation calls alongside the CNA data. We have included analysis of FACS sorted VE-positive cells from the dissociated CDX tumour and have applied NGS to generate both CNA data and specific mutation calls (Fig 5).

In addition, we have included additional analysis of CTCs and circulating tumour DNA (ctDNA) from an extensive stage SCLC patient (Figure 6) where the combined NGS analysis shows 1) VE-Cadherin positive and negative CTC CNA and matched ctDNA CNA are highly related; 2) both CTC and ctDNA show CNA patterns typical SCLC tumours (Fig 6).

The combined data strongly support the tumor origin of the VE-positive cells.

4. How are we sure that VE-positive CTCs are indeed tumor-derived (beyond the conflicting CNA data). The cells have low cytokeratin (CK).

As discussed above in detail, CNA analysis looks at global trends of multiple reads and is unlikely to produce artificial gains and losses, as evidenced by the internal germline controls demonstrating characteristically flat, somatic genomes.

To address the reviewers concerns, we looked to assess further patient samples by HD-SCA. As characterised in Figure 1B(i), VE-Cadherin positive CTCs are an extremely rare population of cells within a cohort of patients. Despite this, we identified an additional patient with DAPI positive, CD45 negative, CK positive and VE-Cadherin positive CTC by HD-SCA. This patient showed common CNA profiles in all CTCs analysed, but lacked consistent CNA aberrations in all 6 WBC controls analysed (Figure 6 and Supplementary Figure 3). In a parallel blood draw, we assessed circulating free tumour DNA (ctDNA), demonstrating an closely similar CNA profile to the single cells analysed by HD-SCA, and with this ctDNA we performed targeted sequencing showing TP53 and RB1 mutations (Supplementary Table 5), hallmarks of SCLC.

Together these data strongly support that VE-Cadherin CTCs have a SCLC origin. Unfortunately we were unable to collect a ctDNA sample from the patient in the original submission analysed by HD-SCA, so were unable to further characterise this patient. As such, the question of sub-clonal populations could not be addressed although it is extremely interesting and subject of further study in the laboratory out of the scope of this manuscript. We have amended and curtailed our reporting throughout the manuscript to focus solely on tumour origin.

5. The quality of the CTC imaging is poor. How is VE cadherin expression quantitated? All cells shown in Figure 1A seem to have positive CD45 staining (a marker of hematopoietic cells). In fact all stains are positive for all immunofluorescence stains: the authors should present quantitative data on immunofluorescence scoring, controls and criteria. The Y axis in figure 1a is not labeled: are these different cells from different patients? In addition, instead of showing a few selected cells, they should show a low resolution image of a filter or epic slide, so that background signal in neighbouring cells can be assessed.

We thank the reviewer for their comments. The imaging and subsequent analysis of ISET filters is technically challenging. Cells can become lodged within pores leading to auto-fluorescent artefacts, making quantitative analysis not feasible.

To address concerns of the reviewer, we have re-generated Figure 1 at much higher resolution, including examples of WBCs and representative CTC types, labelling each panel as requested.

In the original figure, greyscale images only were included, with multiple cell types shown in each panel. We apologise if this mislead the reviewer to think all cells stained for all markers. To confirm, CTCs are defined as DAPI positive and CK positive CD45 negative. We have included single cell examples of all cell types studied and furthermore included an image gallery (Figure 1A and Supplementary Figure 1) to further demonstrate the CTC populations seen.

6. The quality of the staining of tumor vascular structures is poor (Figure 2a). Better resolution is required. There seems to be purple material throughout the tumor slide, rather than a discrete Vessel-like structure (is it background staining?).

We thank the reviewer for their comment. We have now included a higher resolution image in revised Figure 2a demonstrating that PAS structures are not acellular, but rather are lined with histologically typical SCLC cells as demonstrated by the small nucleus and scant cytoplasm. These morphological features would be lost using just CD31 and PAS, requiring the additional staining with haematoxylin counter staining.

7. A key point is that the vascular lining cells of human xenografts are human, rather than mouse-derived. There are human-specific antibodies available to distinguish human from mouse cells,that could make that case much more strongly and convincingly.

We thank the reviewer for highlighting the importance of VM vessels having a human origin. We apologise for not sufficiently clarifying that all studies were carried out using species specific antibodies. This point has now been clarified in the methods and wherever appropriate in the text.

Overall our data conclude that VM vessels in SCLC, identified with human specific antibodies, have CNA profiles consistent with human tumour origin. The evidence is that in CDX tissue, VM vessels were laser micro-dissected based on human anti-VE-Cadherin staining and PAS positive/mouse specific CD31 negative. The resultant genomic analysis of these cells was clearly human derived, with mouse reads accounting for 0-5% of total reads and removed bioinformatically from further analysis. Furthermore, dissociated CDX3 cells labelled with the same human anti-VE-Cadherin antibody and sorted by FACS, excluding mouse cells with anti-mouse MHC1 (new figure 5) had a typical SCLC CNA profile. Targeted sequencing demonstrated that FACs sorted population's harboured the TP53 mutation seen in this CDX model (1), consistent with SCLC.

7. The authors imply but do not explain a VM score in tumors. How is that derived? If VM structures are present in 9/10 xenograft models, how is the score applied. How quantitative is the score, and is the single negative tumor totally negative? Are there other tumor type xenografts in which VM structures are absent (ie negative control)?

We apologise if this description was not clear in the methods. In the manuscript, VM ratio is defined in the methods and results as VM vessels as a percentage of total vessels. Some instances described VM score, which is the same measure, this is now standardised throughout, and we apologise for this lack of clarity. VM score was explained in detail in the methods, we have stated this more clearly when first describing these results (page 6 lines 9-10).

8. Just like VE-CTCs with different CNA compared with tumor CTCs, it is concerning that VM-low regions of tumor xenografts have different genetic CNA than VM-high regions. Is this true for all cases and how does the tumor section CNA relate to the CNA from matched CTCs? Are these consistent within individual patients? Across different patients, it would be strange if in every case, vasculogenic tumor cells originate from a separate tumor precursor that can be easily separated from the bulk tumor cells simply by their different CNA. Again, this calls into question the accuracy of the single cell CNA data and the possibility of technical artifacts.

The primary aim of the NGS analysis we have presented was to demonstrate that both VM-high and VM low cells are of tumour cells and the data we now present more strongly support this contention (Figure 4, Figure 5, Supplementary Table 3 and 4).

In addition, not surprisingly, we have also seen evidence of tumour heterogeneity with subclones picked out by laser dissection (Figure 4) but it is not our contention or within the scope of this publication to suggest a common VM related CNA across patients, we reworded sections in revised manuscript to reflect this (Page 7 line 10-page 9 line 2).

As discussed above, artificial global CNA alterations are unlikely due to multiple reads across the genome being assessed. VM is a rare phenomenon, and in depth study of heterogeneity and VM will require much further study. As such, with this data we have focussed on the human origin of VM vessels and performed targeted sequencing and supported our arguments with FACs sorted cells (Figures 4 Figure 5 Supplementary Table 3 and Supplementary table 4). We have amended our arguments accordingly in the Discussion page 14 line 4-17).

9. The authors conclude that vascular mimicry can be good or bad in that it is associated with a worse clinical prognosis, but appears to reduce tumor hypoxia and improve drug access. These are interesting concepts, but they need to be based on more solid primary data.

We do conclude that VM has a number of potential impacts on tumour behaviour by way of discussing our data. We now show that there is increased cisplatin delivery to VM proficient xenografts and add new in vivo data (new Figure 7, 8, 9 and Supplementary Figure 4 and 5) showing that despite this, cisplatin efficacy is decreased (more drug delivery but less response). VM as a cisplatin resistance mechanism(s) is consistent with the VM associated 'stem cell plasticity' and the precise details of how this is mediated at the molecular level is the subject of on-going and future research and beyond the scope of this manuscript.

Reviewer #3 (vascular mimicry expert)

Metcalf et al in the manuscript entitled "Vaculogenic Mimicry in Samll Cell Lung Cancer" provided solid evidence for the first time that a subpoluation of VE-cadherin-expressing CTCs exists, which drives VM in vivo in the CDX model, and that a VE-cadherin-expression tumor subset exhibits VM capacity in patient samples, which correlates inversely with disease survival. The findings are exciting and significant, because they provide biological rationale for developing therapeutic strategies targeting VM in the treatment of SCLC. The experimental logical and scientifically sound. The approaches and statistical analyses used are appropriate. The analyses of patient-derived materials/ cells as well as the use of biologically relevant animal models significantly strengthen the study. Only minor comments are provided below:

1. **Abbreviations are not spelled out at first mentioning, which makes it difficult to follow the manuscript.**

We thank the reviewer for their comments. We have amended the document throughout to ensure abbreviations are clarified in the main text, with additional improvements to figures and figure legends to ensure abbreviations are suitably explained here also.

2. **How are limited vs. extensive stage diseases defined?**

Definitions of limited stage and extensive stage have been included in the introduction (Page 3 line 2-5). We have standardised nomenclature throughout when describing extensive stage patients and limited stage patients, and improved figure legends to highlight these where relevant (Figure 1B).

3. **The clonality of the VE-cadherin-non-expressing but not the VE-Cadherin-expressing, CTC population as revealed by the CNA analyses is intriguing. It would be helpful to discuss further the significance of this observation and how it relates to disease progression.**

We agree with the reviewer that the sub-clonal populations are of great interest. However, we agree with the reviewers that these data may be too preliminary for publication at this time and subclonal evolution as pertains to VM is the subject of a follow up study. At this time, we agree more in depth study is required, focussing solely on the question of tumour heterogeneity and VM. As such we have focussed on our primary intention, the demonstration that VM vessels are of a human SCLC origin and that VM has functional significance in SCLC.

4. Fig.1A: Additional labeling to highlight indiviual cell type illustrated in the panels; e.g. WBC, VE-Cadherin+ CTC, VE-cadherin- CTC, would be helpful 5. Fig. 1B: What do the 'E's" and 'L's" on top of the graph mean?6. Fig. 2B: It is not clear if nuclear counter stain was used as the tumor cells surrounding the PAS+ channels are difficult to appreciate

Again we thank the review for his relevant observations, and have amended figures accordingly. Figure 1 and Figure 6 have examples of all cell types seen, including WBC and CTCs labelled accordingly.

We have included a cell gallery as Supplementary Figure 1, to show a greater number of CTCs seen across the patients analysed by ISET filtration. Figure 1B has been amended to include a figure legend fixed to explain E and L relate to extensive stage disease and limited stage disease respectively. Finally for Figure 2B, nuclear staining was not included due to difficultly in assessing vessels. Figure 2A demonstrates nuclear counterstain showing vessel lined cells. Updated figures now included at high resolution to clarify this concern.

References

1. Hodgkinson CL, Morrow CJ, Li Y, Metcalf RL, Rothwell DG, Trapani F, et al. Tumorigenicity and genetic profiling of circulating tumor cells in small-cell lung cancer. Nat Med. 2014 Aug;20(8):897-903. PubMed PMID: 24880617. eng.
2. Wagenblast E, Soto M, Gutierrez-Angel S, Hartl CA, Gable AL, Maceli AR, et al. A model of breast cancer heterogeneity reveals vascular mimicry as a driver of metastasis. Nature. 2015 Apr 16;520(7547):358-62. PubMed PMID: 25855289.
3. Maniotis AJ, Folberg R, Hess A, Seftor EA, Gardner LM, Pe'er J, et al. Vascular channel formation by human melanoma cells in vivo and in vitro: vasculogenic mimicry. Am J Pathol. 1999 Sep;155(3):739-52. PubMed PMID: 10487832.
4. Hendrix MJ, Seftor EA, Meltzer PS, Gardner LM, Hess AR, Kirschmann DA, et al. Expression and functional significance of VE-cadherin in aggressive human melanoma cells: role in vasculogenic mimicry. Proc Natl Acad Sci U S A. 2001 Jul 3;98(14):8018-23. PubMed PMID: 11416160. eng.

5. Howlader N, Noone A, Krapcho M, Garshell J, Miller D, Altekruse S, et al. SEER Cancer Statistics Review, 1975-2012, National Cancer Institute. Bethesda, MD, based on November 2014 SEER data submission, posted to the SEER web site, April 2015. http://seer.cancer.gov/csr/1975_2012/2015.
6. Hosokawa M, Kenmotsu H, Koh Y, Yoshino T, Yoshikawa T, Naito T, et al. Size-based isolation of circulating tumor cells in lung cancer patients using a microcavity array system. PloS one. 2013;8(6):e67466. PubMed PMID: 23840710. Pubmed Central PMCID: 3696066.
7. Chudziak J, Burt DJ, Mohan S, Rothwell DG, Mesquita B, Antonello J, et al. Clinical evaluation of a novel microfluidic device for epitope-independent enrichment of circulating tumour cells in patients with small cell lung cancer. The Analyst. 2016 Jan 21;141(2):669-78. PubMed PMID: 26605519.
8. Girotti MR, Gremel G, Lee R, Galvani E, Rothwell D, Viros A, et al. Application of Sequencing, Liquid Biopsies, and Patient-Derived Xenografts for Personalized Medicine in Melanoma. Cancer discovery. 2016 Mar;6(3):286-99. PubMed PMID: 26715644.
9. Rothwell DG, Smith N, Morris D, Leong HS, Li Y, Hollebecque A, et al. Genetic profiling of tumours using both circulating free DNA and circulating tumour cells isolated from the same preserved whole blood sample. Molecular oncology. 2016 Apr;10(4):566-74. PubMed PMID: 26639657. Pubmed Central PMCID: 4834815.

Reviewers' comments:

Reviewer #1 (Remarks to the Author):

The authors have adequately addressed my comments.

Reviewer #2 (Remarks to the Author):

The manuscript is improved, and the most confusing claim, that CTC VE positive cells constitute a genetically distinct subset of the tumor population has been removed. There are however still some questions that need to be addressed:

1. Given that previous publications have demonstrated the tumor origin of VM vessels within tumors, the novelty of the current study is not clear. One of the main claim to novelty in the previous version had been the concept of subclonal variation, which is now no longer proposed. The primary novelty is the extention to small cell lung cancer of a phenomenon shown by lineage tracing in other tumor types.

2. The authors have added more convincing images of the CTC staining, but the VE Cadherin staining appears to be nuclear in Fig 1A (in both cell shown). That causes some concern about specificity of staining. Should VE Cadherin not be membrane staining?

3. The proposed therapeutic implications still don't make too much sense. The authors seem to claim that CTC-VE cells are more resistant to platinum, so that the increased drug delivery resulting from their creation of vascular access is counteracted by their intrinsic cellular resistance. However, these cells constitute a small subset of the tumor mass and there is no data to show that following platinum treatment, CTC-VE cells are enriched due to their drug resistance. This data is relatively borderline in terms of effect size, and the message is confusing. I would recommend that the authors delete this section since it detracts from the credibility of the study.

4. The authors mention that the results were shown in only 1/8 SCLC cell lines tested. It would seem important to have at least one other cell line that shows the same effect.

Reviewer #3 (Remarks to the Author):

The authors have substantially revised the manuscript to address comments raised by in the initial review. As a result, the resubmitted version of the manuscript is significantly improved in

many aspects, including clarity, presentation, and data quality. The additional experiments performed also strengthened/enhanced the conclusions drawn. I have no further concern and believe that it is suitable for publication at its current form.

Reply to Reviewers' comments:

We thank the Reviewers and Editor for their comments in the first round review that have assisted us in improving our manuscript. We were pleased to see the Reviewers think the manuscript has been improved as a result of our amendments. We have addressed each new comment point by point below, but in summary, we have improved the images presented in Figure 1 and added text extending and clarifying the discussion of the xenograft data presented in Figure 9 on page 15 of the second revision of our manuscript.

Point by Point reply

Reviewer #2 (Remarks to the Author):

The manuscript is improved, and the most confusing claim, that CTC VE positive cells constitute a genetically distinct subset of the tumor population has been removed. There are however still some questions that need to be addressed:

1. Given that previous publications have demonstrated the tumor origin of VM vessels within tumors, the novelty of the current study is not clear. One of the main claim to novelty in the previous version had been the concept of subclonal variation, which is now no longer proposed. The primary novelty is the extention to small cell lung cancer of a phenomenon shown by lineage tracing in other tumor types.

To briefly reiterate, we feel the key novel findings are; demonstration for the first time a VM phenotype in CTCs; identified VM in SCLC, genomic proof that VM vessel cells are of tumour origin and in SCLC, VE-Cad is required for VM and demonstrate functional roles for VM with respects to tumour growth kinetics, drug delivery and chemotherapy response.

2. The authors have added more convincing images of the CTC staining, but the VE Cadherin staining appears to be nuclear in Fig 1A (in both cell shown). That causes some concern about specificity of staining. Should VE Cadherin not be membrane staining?

We thank the Reviewer for their comment. In Figure 1, SCLC CTCs were captured (by vacuum suction) onto ISET filters and these are inherently quite difficult to image. SCLC cells are also classically small, with a large nucleus and scant cytoplasm. Having said this, we can see what Reviewer 2 is getting at if one looked only at the 2 VE-Cadherin positive cells in Figure 1A (top panel). We have replaced VE-Cad positive CTCs for Figure 1 which is (and was in the original revision 1) complemented with 10 additional VE-Cad positive CTCs in Supplementary Figure 1. The replaced CTCs in Figure 1 show one that is at top end of the range of CTC sizes, with VE-Cadherin staining throughout and certainly not just nuclear, and another of a more typical size, where despite the scant cytoplasm there is VE-Cadherin staining not confined to the nucleus. The

CTCs in supplementary Figure 1(ii) also clearly show that VE-Cad staining is not predominantly nuclear. Moreover, we contest this reviewer's assertion of non-specific staining on the basis that if it were non-specific, it is unlikely that it would be so rare and variable a population across so many independent samples.

3. The proposed therapeutic implications still don't make too much sense. The authors seem to claim that CTC-VE cells are more resistant to platinum, so that the increased drug delivery resulting from their creation of vascular access is counteracted by their intrinsic cellular resistance. However, these cells constitute a small subset of the tumor mass and there is no data to show that following platinum treatment, CTC-VE cells are enriched due to their drug resistance. This data is relatively borderline in terms of effect size, and the message is confusing. I would recommend that the authors delete this section since it detracts from the credibility of the study.

In this proof of concept xenograft model it is impossible to look at enrichment because in the parental H446 xenografts, all cells express VE-Cad. In the VE-Cad knock-down xenografts, Figure 7C(iii) shows lack of detectable VE-Cadherin in all the tumour lysates from all 6 animals, therefore we could not look for enrichment here of a drug resistant subset of VE-Cad positive cells either. We cannot therefore provide, data on enrichment of VM competent VE-Cadherin expressing cells due to drug resistance. We suggest based on our TMA and CTC clinical data (Figures 1 and 2) that VM competent cells are rare in SCLC patients at baseline. The experiment this reviewer seems to want is a comparison of VE-Cad expressing VM competent cells at baseline and at disease progression with drug resistance. Whilst this is a good suggestion, there are 2 logistical problems. First and foremost, patients do not provide tumour biopsies at disease progression, where there are no current therapeutic options. Second, if we were to approach this question using CTCs as a surrogate to evaluate VE-Cad expression and hence VM in tumour, we would need a prospective collection of sufficient matched CTC samples at baseline and again at disease progression, retesting the patient for chemosensitivity at progression. This CTC study could take us up to a year, assuming patients were fit enough for chemotherapy rechallenge (they are often not). Lastly, as SCLC is so genomically unstable, there are almost certainly multiple mechanisms of chemotherapy resistance.
For the reasons highlighted above, we respectfully decline to address this experimentally. We have however, added new text in the discussion section to clarify that the xenograft data is a proof of concept, that SCLC cells expressing VE-Cadherin in an *in vivo* context are more resistant to chemotherapy, than those where VE-Cadherin is not (or minimally) expressed (Page 15, line 4-12). We also highlight that longer term studies are needed to test this hypothesis in clinical samples, beyond the scope of this manuscript (Page 15 Line 16-21).
Finally, we are surprised by this Reviewer's comment that the size effect was borderline. Figure 9A shows quite clearly that xenograft tumour regression occurs after chemotherapy in every animal bearing H446 VE-Cad knock down tumours (green lines), whereas the kinetics of tumour growth for all animals

bearing parental H446 xenografts (pink) is indistinguishable from vehicle treated controls (pink and blue lines).

4. The authors mention that the results were shown in only 1/8 SCLC cell lines tested. It would seem important to have at least one other cell line that shows the same effect.

This point was raised by Reviewer #1 in the first round of review, and we pointed out in our rebuttal that VE-Cad knock down on C8161 melanoma cells had been previously reported by our co-author Mary Hendrix, confirming its functional relevance for uveal melanoma VM. We had previously stated we could not find another SCLC cell line that expressed detectable levels of VE-Cadherin and generate VM like networks *in vitro*. We have now screened a further 4 SCLC cell lines, none express VE-Cadherin. We cannot therefore perform additional experiments using SCLC cell lines We are trying to genetically modify our CDX cell cultures to manipulate VE-Cadherin and interrogate VM, but as stated previously in response to Reviewer #1, this is technically very challenging and at this point in time not robust. We have added to the text this long term goal to modulate VE-Cad expression in CDX once we address the technical challenges of this approach (Page 15, line 16-21)
